# Deciphering Cellodextrin and Glucose Uptake in *Clostridium thermocellum*

**DOI:** 10.1128/mbio.01476-22

**Published:** 2022-09-07

**Authors:** Fei Yan, Sheng Dong, Ya-Jun Liu, Xingzhe Yao, Chao Chen, Yan Xiao, Edward A. Bayer, Yuval Shoham, Chun You, Qiu Cui, Yingang Feng

**Affiliations:** a CAS Key Laboratory of Biofuels, Shandong Provincial Key Laboratory of Synthetic Biology, Qingdao Institute of Bioenergy and Bioprocess Technology, Chinese Academy of Sciences, Qingdao, China; b Shandong Energy Institute, Shandong, Qingdao, China; c Qingdao New Energy Shandong Laboratory, Shandong, Qingdao, China; d Dalian National Laboratory for Clean Energy, Shandong, Qingdao, China; e University of Chinese Academy of Sciences, Beijing, China; f Department of Biomolecular Sciences, The Weizmann Institute of Science, Rehovot, Israel; g Department of Life Sciences and the National Institute for Biotechnology in the Negev, Ben-Gurion University of the Negev, Beer-Sheva, Israel; h Department of Biotechnology and Food Engineering, Technion-Israel Institute of Technology, Haifa, Israel; i Tianjin Institute of Industrial Biotechnology, Chinese Academy of Sciences, Tianjin, China; Kyoto University; Korea Advanced Institute of Science and Technology

**Keywords:** ABC transporter, genetic inactivation, sugar-binding protein, protein structure, cellulosome

## Abstract

Sugar uptake is of great significance in industrially relevant microorganisms. Clostridium thermocellum has extensive potential in lignocellulose biorefineries as an environmentally prominent, thermophilic, cellulolytic bacterium. The bacterium employs five putative ATP-binding cassette transporters which purportedly take up cellulose hydrolysates. Here, we first applied combined genetic manipulations and biophysical titration experiments to decipher the key glucose and cellodextrin transporters. *In vivo* gene inactivation of each transporter and *in vitro* calorimetric and nuclear magnetic resonance (NMR) titration of each putative sugar-binding protein with various saccharides supported the conclusion that only transporters A and B play the roles of glucose and cellodextrin transport, respectively. To gain insight into the structural mechanism of the transporter specificities, 11 crystal structures, both alone and in complex with appropriate saccharides, were solved for all 5 putative sugar-binding proteins, thus providing detailed specific interactions between the proteins and the corresponding saccharides. Considering the importance of transporter B as the major cellodextrin transporter, we further identified its cryptic, hitherto unknown ATPase-encoding gene as *clo1313_2554*, which is located outside the transporter B gene cluster. The crystal structure of the ATPase was solved, showing that it represents a typical nucleotide-binding domain of the ATP-binding cassette (ABC) transporter. Moreover, we determined that the inducing effect of cellobiose (G2) and cellulose on cellulosome production could be eliminated by deletion of transporter B genes, suggesting the coupling of sugar transport and regulation of cellulosome components. This study provides key basic information on the sugar uptake mechanism of *C. thermocellum* and will promote rational engineering of the bacterium for industrial application.

## INTRODUCTION

Lignocellulose biorefineries provide new pathways for the eco-friendly and sustainable production of energy and chemicals (1–4). One of the major bottlenecks for cost-effective production of biofuels and bio-based chemicals is the recalcitrance of lignocellulose, and thus, studies on lignocellulolytic microorganisms are of great interest (5–8).

Clostridium thermocellum (also named Ruminiclostridium thermocellum, Hungateiclostridium thermocellum, and Acetivibrio thermocellus) is a thermophilic, lignocellulolytic, Gram-positive bacterium with great potential to be integrated into various strategies of lignocellulose biorefinery, particularly in consolidated bioprocessing and consolidated bio-saccharification (9–12). *C. thermocellum* produces a multienzyme complex, the cellulosome, which efficiently degrades cellulose into oligosaccharides (13, 14). The intracellular metabolism of carbohydrates in *C. thermocellum* has been extensively investigated and engineered for the production of high-value chemicals (15). However, the process of sugar uptake by *C. thermocellum* has not been comprehensively studied, except for the identification of several putative sugar ATP-binding cassette (ABC) transporters that have been proposed to be responsible for cellodextrin transport (16, 17).

Highly efficient uptake of hydrolyzed substrates is important to cell factories in lignocellulose biorefineries, and sugar transporters are therefore of great interest in the study of lignocellulolytic microorganisms (18). Unlike most cellulolytic fungi and bacteria, which prefer glucose as a carbon source, *C. thermocellum* prefers to utilize oligosaccharides and exhibits poor growth on monosaccharides (19–22). Previously, five putative sugar transporters, denoted A to D and L and belonging to the ABC transport systems group, were identified in the genome of *C. thermocellum*, and the solute-binding lipoproteins (SBP) of these ABC transporters were shown to bind different dextrins by isothermal titration calorimetry (ITC) assays (17). Four SBPs, denoted cellodextrin-binding proteins A to D (CbpA to CbpD), were shown to bind to glucose and/or different lengths of cellodextrins; and one SBP, denoted laminaribiose-binding protein (Lbp), was shown to bind laminaribiose. However, the function(s) of these putative sugar transporters has not been verified *in vivo*.

In this study, we first investigate the function of the five putative sugar transporters by genetic inactivation of each transporter. Surprisingly, the growth phenotypes of these mutants indicated that transporter B is the sole cellodextrin transporter, whereas transporter A is the sole glucose transporter. These results were further supported by both biophysical and structural evidence, which provided the mechanism of substrate specificity of the sugar-binding proteins in these transporters. Considering the importance of transporter B as the sole cellodextrin transporter, we identified the ATPase gene which is missing in the transporter B gene cluster. The correlation between transporter B and cellulosome production was determined by characterizing the transporter B deletion mutant.

## RESULTS

### Genetic identification of key ABC transporters for glucose and cellodextrin.

**FIG 1:**
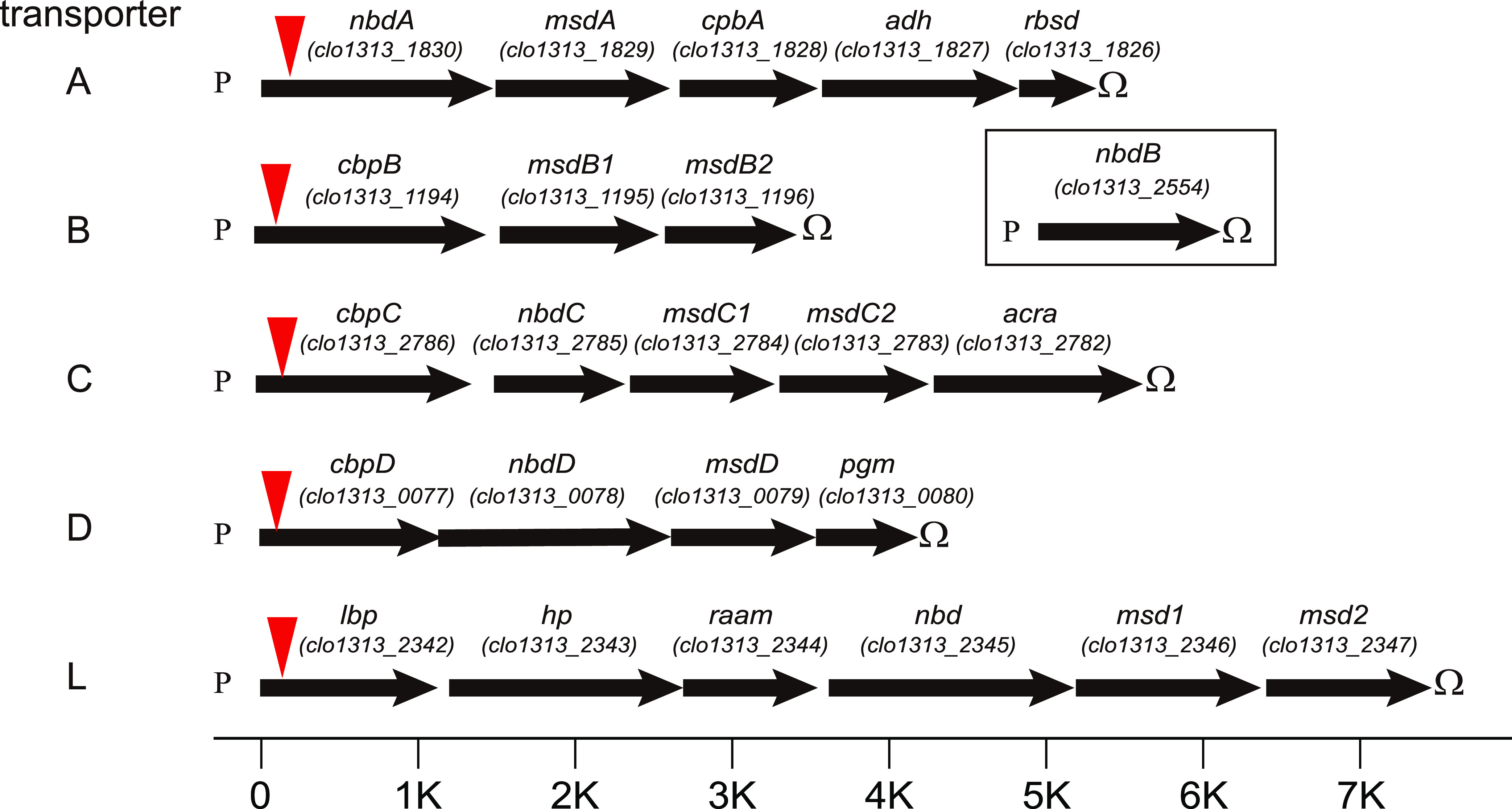
Schematic representation of the genetic organization of the sugar ATP-binding cassette (ABC) transporters in Clostridium thermocellum. The letters P and Ω symbolize potential promoters and rho-independent terminators, respectively. Red triangle indicates intron insertion site. Gene names: *nbd*, nucleotide-binding domain; *msd*, membrane-spanning domain; *cbp*, cellodextrin-binding protein; *adh*, alcohol dehydrogenase; *rsbd*, RbsD/FucU transport protein family; *acra*, multidrug efflux pump subunit AcrA; *lbp*, laminaribiose-binding protein; *pgm*, phosphoglycerate mutase; *hp*, hypothetical protein; *rsam*, radical *S*-adenosylmethionine.

To further verify the function of the putative sugar ABC transporters *in vivo*, we inactivated each transporter gene by using thermotargetrons, which insert a group II intron RNA into the first gene of the operon (Fig. 1). Inactivation mutants of transporters A, C, D, and L (denoted Δ*transporterA*, Δ*transporterC*, Δ*transporterD*, and Δ*transporterL*) were obtained with cellobiose (G2) as the carbon source in the GS-2 medium, while the transporter B mutant (denoted Δ*transporterB*) was obtained with glucose as the carbon source.

**FIG 2:**
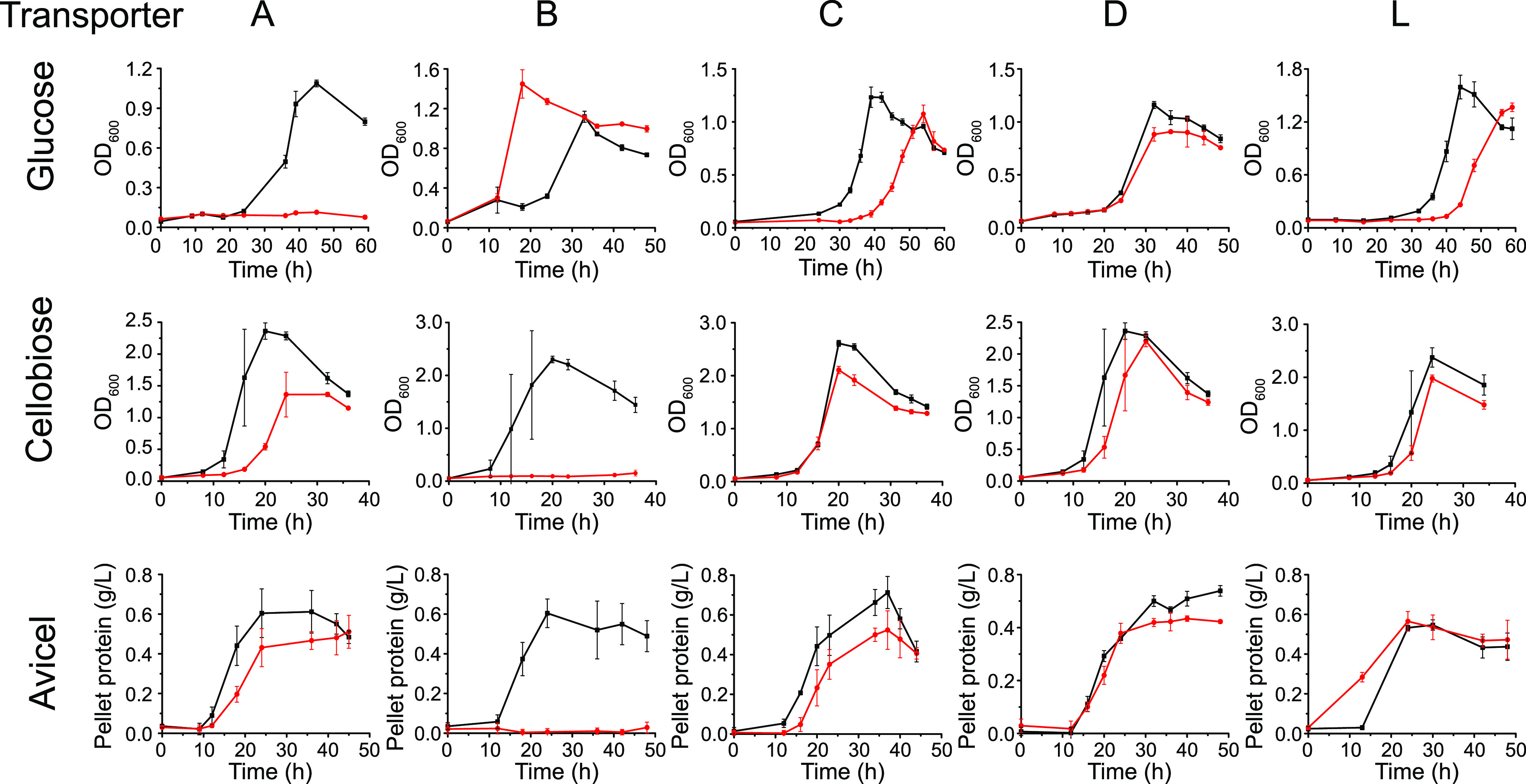
Growth curves of *C. thermocellum* transporter inactivation mutants (red) and wild type (WT) (black) on glucose, cellobiose, and Avicel. Optical density at 600 nm (OD_600_) and the pellet protein were measured on soluble (glucose and cellobiose) and insoluble (Avicel) carbon sources, respectively. All data represent the mean of triplicate cultures and bars indicate ± standard deviation (SD).

We then monitored the growth curves of the mutant and wild-type strains in GS-2 media with glucose, cellobiose, or Avicel (microcrystalline cellulose) as the sole carbon source. Although a previous study (17) showed that Cbp A to D can all bind to cellodextrin and only CbpC binds to glucose, we found in this study that the Δ*transporterA* strain lost the ability to grow on glucose and the Δ*transporterB* strain was unable to grow on cellobiose or Avicel (Fig. 2). Unexpectedly, the Δ*transporterC*, Δ*transporterD*, and Δ*transporterL* strains grew normally on all three substrates (Fig. 2). Considering that thermotargetrons potentially have additional off-target insertions (23, 24), we additionally constructed transporter A and transporter B deletion mutants using seamless genome editing (25). As shown in Fig. S1 in the supplemental material, the growth phenotype of the mutant strains was the same as that of the mutants obtained by thermotargetron inactivation, and the respective phenotype can be restored by using plasmid-based expression of the transporter genes. These results indicated that transporter A is the major glucose transporter and transporter B is the major cellodextrin transporter in *C. thermocellum.*

10.1128/mbio.01476-22.1 FIG S1 Growth curves of wild-type Clostridium thermocellum, transporter A/B deletion mutants by homology recombination (h.r.), and plasmid-based complementation strain on the designated carbon source. Optical density at 600 nm (OD_600_) and the pellet protein were measured on soluble (glucose and cellobiose) and insoluble (Avicel) carbon sources, respectively. All data represent the mean of triplicate cultures and the bars show ± standard deviation (SD). Download FIG S1, PDF file, 0.6 MB. Copyright © 2022 Yan et al. 2022 Yan et al. https://creativecommons.org/licenses/by/4.0/ This content is distributed under the terms of the Creative Commons Attribution 4.0 International license.

### Substrate specificity of the Cbps.

**FIG 3:**
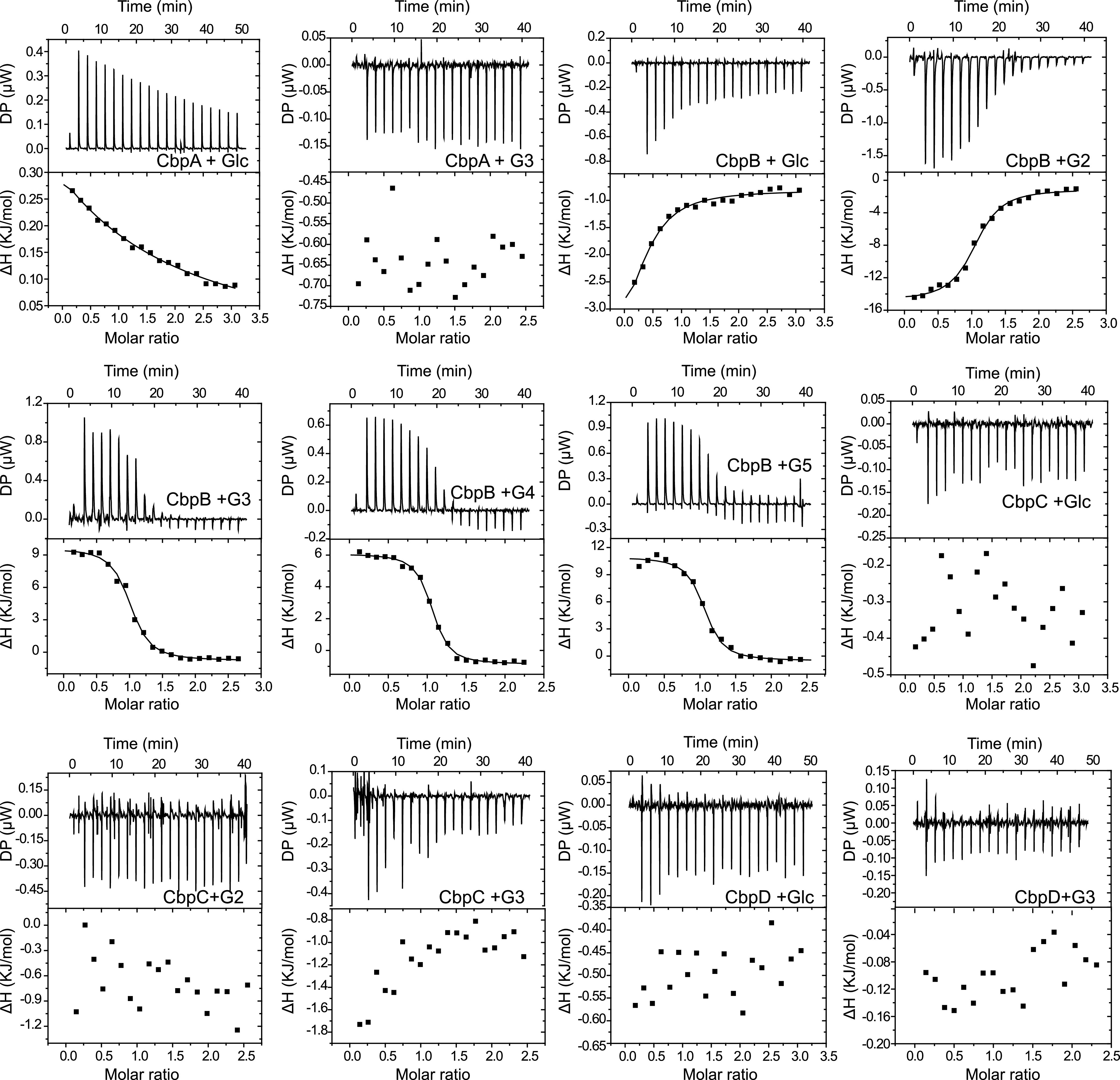
Representative isothermal titration calorimetry (ITC) data for titrations of various carbohydrate ligands with the cellodextrin-binding proteins (Cbps). ITC traces shown on top, integrated binding isotherms shown on the bottom. The Cbp and substrate used are shown in each ITC trace. Glc, glucose; G2, cellobiose; G3, cellotriose; G4, cellotetraose; G5, cellopentaose.

The results of our genetic experiments indicated that transporters A and B are essential for supporting growth on glucose and cellodextrin, respectively. However, previous ITC experiments using SBPs and the appropriate sugars showed that transporters A to D can all bind to cellodextrins, whereas only CbpC can bind to glucose (17). To further address the discrepancy between our genetic data and previous ITC data in the literature, we performed ITC and NMR experiments to determine the interactions between Cbps and various sugars, including cellodextrins (G2 to G5), glucose (G1), and xylose. The ITC experiments showed that among all of the tested substrates, only CbpA binds to glucose, with a dissociation constant (*K_D_*) of 240 μM (Fig. 3, Table 1). In agreement with the ITC data, NMR titrations showed that CbpA binds glucose but not cellobiose or cellotriose (Fig. S2). CbpB showed high affinities for all of the tested cellodextrins (G2 to G5), with the highest affinity for cellotetraose (*K_D_* = 0.562 μM) (Fig. 3, Table 1). Interestingly, CbpB also appeared to interact with glucose, but the data could not be fitted to obtain reasonable N and *K_D_* values. Furthermore, NMR titration showed that CbpB can bind to cellobiose but not to glucose (Fig. S2). For CbpC and CbpD, no interaction was detected for all of the tested carbohydrate substrates in both ITC and NMR experiments (Fig. S2), suggesting that transporters C and D are not responsible for glucose or cellodextrin transport. Thus, the ITC and NMR results are consistent with the genetic experiments, confirming the essential roles of transporters A and B for glucose and cellodextrin transportation, respectively.

**TABLE 1:** Thermodynamic parameters determined by ITC measurements^*a*^

Sbp	Substrate	N	*K_D_* (μM)	*K*_a_ (×10^5^ · M^−1^)	ΔH (kJ/mol)	ΔG (kJ/mol)	TΔS (kJ/mol)
CbpA	Glucose	1.06 ± 0.07	240 ± 95	0.042 ± 0.016	1.71 ± 0.36	−21.0	22.7
CbpB	Cellobiose	1.03 ± 0.02	1.89 ± 0.36	5.29 ± 0.99	−13.7 ± 0.4	−33.2	19.5
	Cellotriose	0.97 ± 0.02	1.17 ± 0.23	8.55 ± 1.69	10.4 ± 0.3	−34.5	44.9
	Cellotetraose	1.02 ± 0.08	0.56 ± 0.08	17.8 ± 2.5	6.95 ± 0.11	−36.3	43.3
	Cellopentaose	1.02 ± 0.01	0.67 ± 0.15	14.9 ± 3.3	11.5 ± 0.3	−35.9	47.3
	Laminaribiose	0.98 ± 0.04	10.7 ± 2.8	0.93 ± 0.25	4.57 ± 0.42	−28.9	33.4

a ITC, isothermal titration calorimetry; N, number of binding site; *K_D_*, dissociation constant; *K*_a_, absorption rate constant; ΔH, change in enthalpy; Cbp, cellodextrin-binding protein.

10.1128/mbio.01476-22.2 FIG S2 Nuclear magnetic resonance (NMR) titration of cellodextrin binding proteins (Cbps) with various saccharides. Overlaid ^1^H-^15^N HSQC spectra of each ^15^N-labelled Cbp with (red) and without (black) the respective saccharide are shown, and significant peak shifts (spectral regions indicated by magenta dashed ellipse) can be observed for those with detectable interactions (CbpA with glucose, CbpB with cellobiose and cellotriose). The molar ratios of Cbp and saccharides are shown at the top of each spectrum. Download FIG S2, PDF file, 1.1 MB. Copyright © 2022 Yan et al. 2022 Yan et al. https://creativecommons.org/licenses/by/4.0/ This content is distributed under the terms of the Creative Commons Attribution 4.0 International license.

### Overall structures of the Cbps.

To identify the carbohydrate ligand-binding mechanism of the different Cbps, we successfully solved the crystal structures of unliganded CbpA, CbpB, CbpC, and CbpD, as well as the complex structures of CbpA with glucose and CbpB with cellobiose, cellotriose, cellotetraose, and cellopentaose (Table S1). Similar to the typical SBP fold, all of the Cbps adopted two-domain structures connected by three hinge regions, with the buried ligand-binding site between the two domains. Although each Cbp was crystallized in the presence of various carbohydrates, including glucose, xylose, cellobiose, cellotriose, cellotetraose, and cellopentaose, only those Cbps and ligands showing interactions in the ITC experiments were successfully crystallized in the liganded forms.

10.1128/mbio.01476-22.6 TABLE S1 Crystallographic data collection and refinement statistics. Download Table S1, PDF file, 0.2 MB. Copyright © 2022 Yan et al. 2022 Yan et al. https://creativecommons.org/licenses/by/4.0/ This content is distributed under the terms of the Creative Commons Attribution 4.0 International license.

### Structure of CbpA.

**FIG 4:**
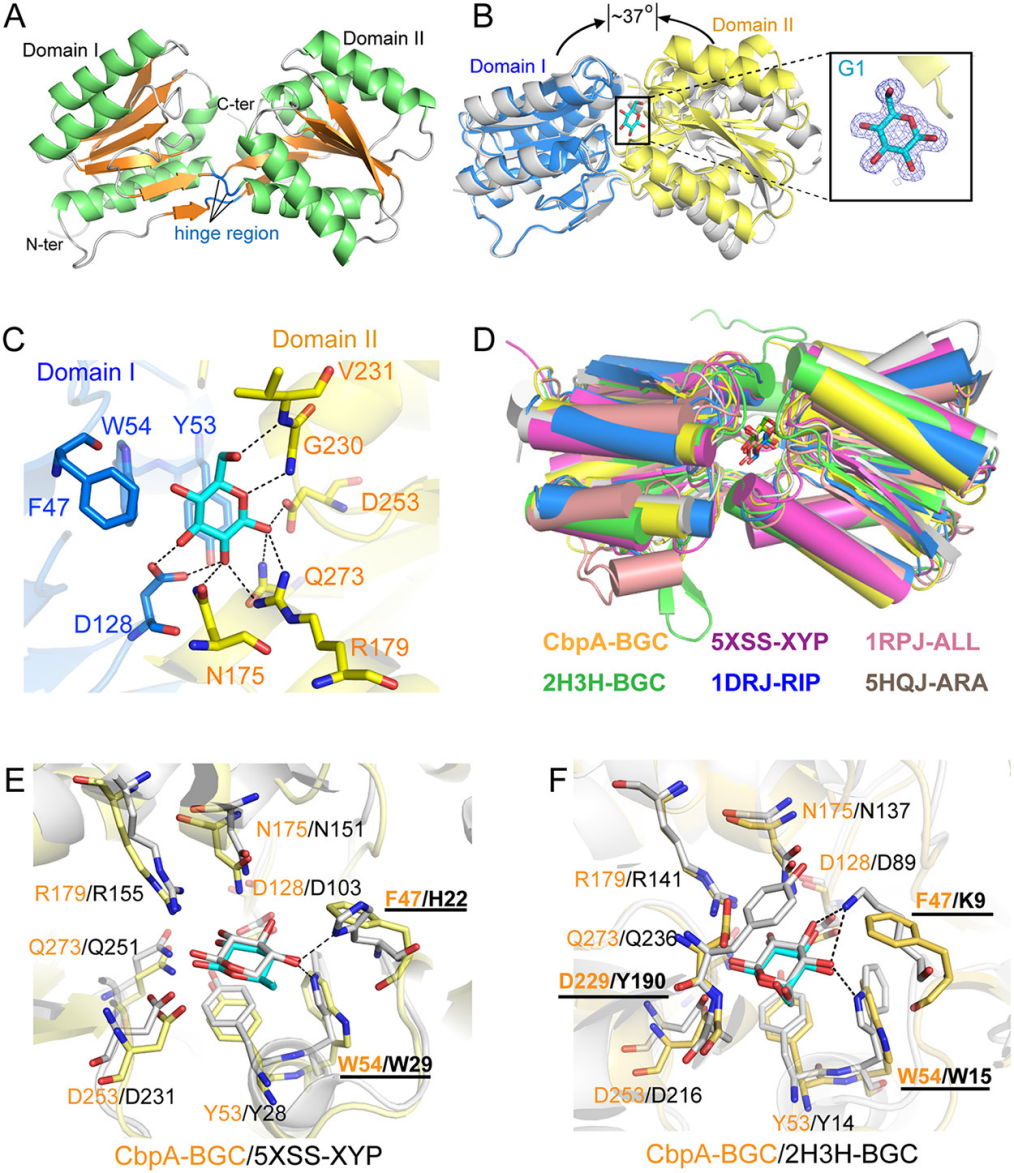
Structure of cellodextrin-binding protein A (CbpA). (A) Unliganded structure. α-Helices and β-strands are colored in green and orange, respectively. The hinge region includes three loops (colored blue) which connect the two domains. (B) Conformational change of CbpA upon binding of glucose. The CbpA-glucose complex structure (domain I in blue and domain II in yellow) is overlaid with the unliganded CbpA (gray) by the superimposition of domain I. In the inset, the glucose (G1) in the complex structure is shown in cyan sticks, and the 2mFo-DFc densities for the glucose are contoured in blue at 1.0 σ. (C) Interactions between glucose and surrounding residues of CbpA. Potential hydrogen bonds are shown as dashed lines. (D) Structural comparison of CbpA and other monosaccharide-binding proteins. The Protein Data Bank (PDB) numbers and monosaccharides (BGC, glucose; XYP, xylose; RIP, ribose; ALL, allose; ARA, arabinose according to identifiers from the PDB Chemical Component Dictionary) are indicated with the same color of proteins. (E and F) Detailed comparison of the interactions between the protein and monosaccharide for CbpA (yellow with orange labels) and two other monosaccharide-binding proteins (xylose-binding protein XylFII in panel E and glucose-binding protein tmGBP in panel F, gray with black labels). Additional hydrogen bonds in the structures of XylFII-xylose (PDB ID 5XSS) and tmGBP (PDB ID 2H3H) are shown as dashed lines. Labels of non-conserved binding residues are underlined.

The crystal structures of both unliganded and glucose-liganded CbpA were determined to 2.10 and 1.85 Å resolution, respectively (Table S1). Unambiguous electron density for β-d-glucose was observed in the CbpA/Glc complex, indicating that the ligand molecule was bound in a single chair conformation. According to the “Venus-flytrap” mechanism (26, 27), i.e., ligand binding causes a dramatic conformational change of two-domain SBPs from an open to a closed state. Hence, the structure of unliganded CbpA assumes an open conformation (Fig. 4A), while CbpA in complex with glucose adopts a closed-conformation, resulting in approximately 37° rigid-body rotation with respect to the unliganded CbpA structure (Fig. 4B). The glucose is bound via hydrogen-bonding interactions and CH-Pi interactions with CbpA (Fig. 4C). Furthermore, the binding pocket of CbpA is distinctly small for the accommodation of a monosaccharide, and no additional space appears available for the binding of larger cellodextrins in CbpA, thus explaining the functional specificity of transporter A.

We searched similar structures in the Protein Data Bank (PDB) using the BLAST (28) and Dali servers (29) with the amino acid sequence and structure of CbpA, respectively. The top hits of both searches were monosaccharide-binding proteins of ABC transporters for the transport of glucose, xylose, arabinose, ribose, etc. Structural comparisons with some of them (30–34) showed that they could be superimposed well in the overall structure (Fig. 4D). However, some hydrogen bonds between residues with the ligand were missing in CbpA compared to the reference structures. For example, in XylFII from Clostridium beijerinkii and tmGBP from Thermotoga maritima, H22 and K9 form hydrogen bonds with O4 of the ligand, respectively. However, in *C. thermocellum* CbpA, a phenylalanine residue (F47) occupies the corresponding location and thus cannot form a similar hydrogen bonding interaction with the substrate. In addition, W54 in CbpA adopted a conformation that is also inconsistent with hydrogen bond formation (Fig. 4E). Furthermore, Y190 in tmGBP forms aromatic ring stacking with glucose, as opposed to D229 which occupies the same position in CbpA (Fig. 4F). The reduced interactions between sugar and protein in CbpA are consistent with the fact that the affinity between glucose and CbpA (*K_D_* of 240 μM) is much lower than that of the other binding proteins (*K_D_* of 1 μM or less).

### Structure of CbpB.

**FIG 5:**
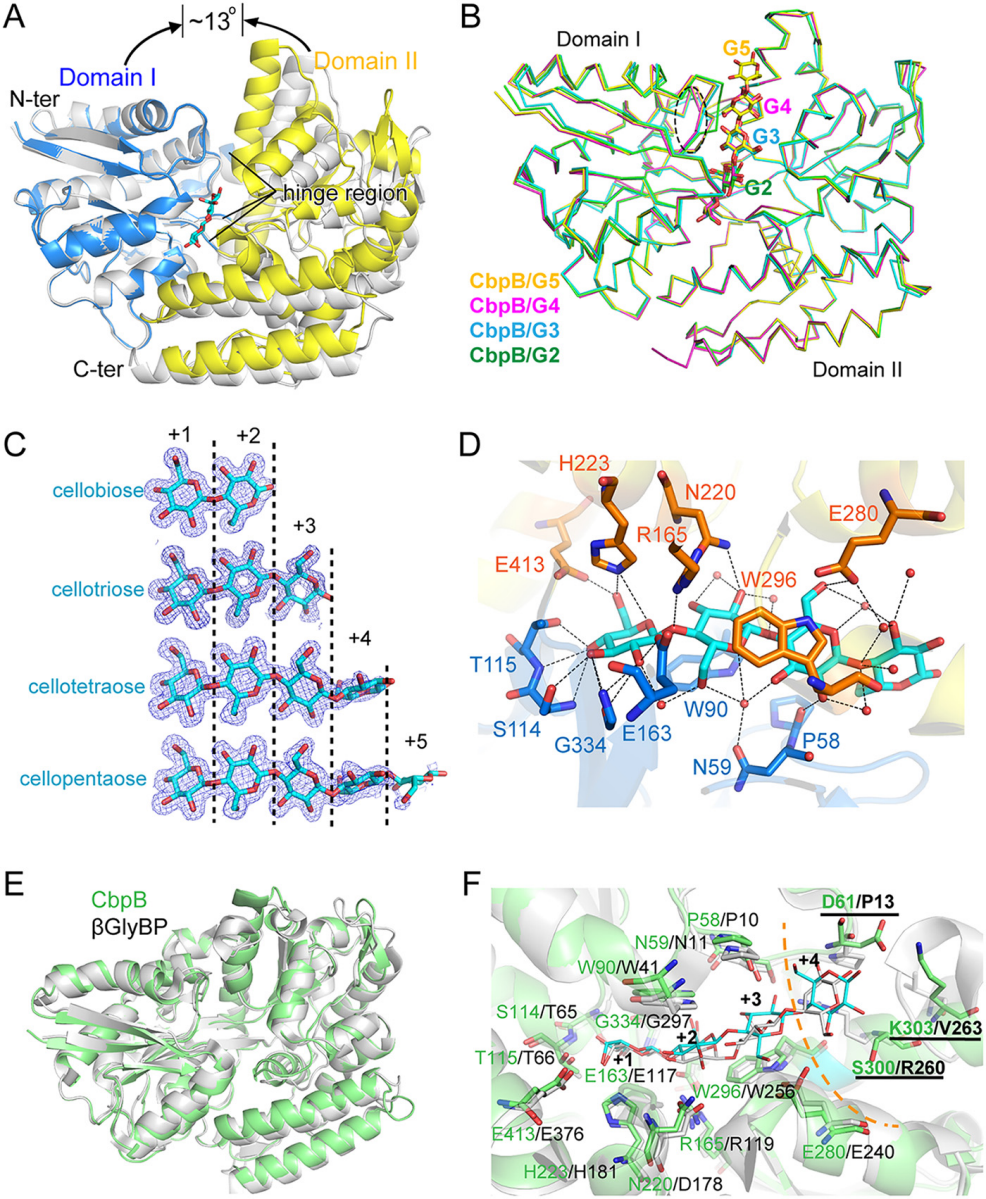
Structure of CbpB. (A) Conformational change of CbpB upon binding of cellobiose (G2). The CbpB-G2 complex structure (domain I in blue and domain II in yellow) was overlaid with the unliganded CbpB (gray) by the superimposition of domain I. (B) Superimposed backbone traces of CbpB-cellodextrin complexes. CbpB-G2, green; CbpB-G3, cyan; CbpB-G4, magenta; CbpB-G5, yellow. (C) Conformations of cellodextrins in the CbpB-cellodextrin structures. The 2mFo-DFc densities for the cellodextrins are contoured in blue at 1.0 σ. (D) Residues involved in cellotetraose binding of CbpB. Cellotetraose is shown as cyan sticks and residues from domains I and II are shown as orange and blue sticks, respectively. Water molecules are shown as red spheres. Potential hydrogen bonds between cellotetraose and CbpB are shown as dashed lines. (E and F) Comparison between the structures (E) and sugar-binding sites (F) of CbpB (green) and the β-glucosides-binding protein (βGlyBP) from Thermus thermophilus HB8 (gray). The cellotetraose molecules complexed with CbpB and βGlyBP are shown as cyan and gray sticks, respectively. The dashed orange curve separates conserved +1 to +3 subsites (residues with plain labels) and the non-conserved +4 and +5 subsites (residues with underlined labels).

The unliganded CbpB structure and the complex structures of CbpB with cellodextrins (G2 to G5) were determined to 1.70 to 2.00 Å resolution (Table S1). A hinge motion was observed between the unliganded CbpB and its complexed structures, and the rigid body rotation angle was ~17^°^ (Fig. 5A). The complex structures of CbpB with G2 to G5 could be superimposed well (Fig. 5B), with a root mean square deviation (RMSD) of 0.15 to 0.51 Å. CbpB exhibited a long substrate-binding pocket between the two domains, and the glucose residues at subsites +1 to +3 showed unambiguous electron densities (Fig. 5C), indicating tight ligand fixations at these subsites. The electron densities for the glucose residue at subsite +4, however, were somewhat ambiguous, and those at subsite +5 were hardly observed, indicating the flexibility of the glucose residues at these subsites. The fifth glucose residue in the CbpB-G5 complex indeed showed a problematic conformation in validation, resulting from insufficient electron density. A large number of residues at subsites +1 to +4 are involved in the extensive hydrogen-bond network formed with the cellodextrin hydroxyl groups in CbpB (Fig. 5D). Furthermore, W90 and W296 lay parallel with the glucose residues at subsites +2 and +3, respectively, and formed aromatic stacking interactions with the ligand. The fifth glucose residue extended outside the protein and had no significant direct interaction with the protein.

After we determined the structure of CbpB, we found that structures of a β-glucosides-binding protein (βGlyBP) from Thermus thermophilus HB8 with 39% sequence identity had been reported in 2020 (35). βGlyBP can bind to different types of oligosaccharides with various glycosidic linkages and 2 to 5 glucosyl units (35). Structural comparison of these two proteins showed that the overall structures could be superimposed well (Fig. 5E). The residues at subsites +1 to +3 were conserved and showed similar conformations, whereas some variations existed at subsites +4 and +5 for the two proteins (Fig. 5F). The structural similarity confirms that CbpB is a conserved cellodextrin-binding protein. The structures of the CbpB-cellodextrin complex revealed that the longer cellodextrins had more interactions with protein until DP 4, which is consistent with its highest binding affinity with cellotetraose (Table 1).

Considering that βGlyBP from Thermus thermophilus can bind to laminaribiose (35), we suspected that CbpB also serves as a laminaribiose-binding protein. ITC experiments indicated that CbpB can indeed bind to laminaribiose, albeit with a lower affinity compared to cellobiose (Fig. S3A, Table 1). We also successfully determined the crystal structure of the CbpB-laminaribiose complex (Fig. S3B, Table S1). Laminaribiose was bound in the same binding site as cellobiose with the same hydrogen-bonding pattern at subsite +1 but a different pattern at subsite +2 (Fig. S3C). These data suggest that transporter B can also serve as a laminaribiose transporter. However, the mutant Δ*transporterB* strain can still grow well with laminaribiose as the sole carbon source (Fig. S3D), suggesting that transporter B is not the sole laminaribiose transporter in *C. thermocellum*.

10.1128/mbio.01476-22.3 FIG S3 Structure of CbpB-laminaribiose. (A) Isothermal titration calorimetry (ITC) result for titrations of laminaribiose into laminaribiose-binding protein (Lbp). (B) Structure of CbpB in complex with laminaribiose. The α-helices and β-strands are colored in green and orange, respectively. Laminaribiose is shown as cyan sticks. In the inset, the 2mFo-DFc densities for the laminaribiose are contoured in blue at 1.0 σ. (C) Comparison of the sugar-binding sites of CbpB in complex with laminaribiose (green) and cellobiose (gray). (D) Growth curves of the Δ*transporterB* (red) and wild-type (black) strains on medium with laminaribiose as carbon source. Download FIG S3, PDF file, 2.1 MB. Copyright © 2022 Yan et al. 2022 Yan et al. https://creativecommons.org/licenses/by/4.0/ This content is distributed under the terms of the Creative Commons Attribution 4.0 International license.

### Structure of other potential sugar-binding proteins.

The unliganded CbpC and CbpD structures were determined to 2.00 and 1.50 Å resolution, respectively (Table S1) (Fig. S4A and C). A Dali search using the CbpC structure revealed similarities with several sugar- and other solute-binding proteins of ABC transporters (Fig. S4B). However, the expected substrate-binding pocket between domain I and domain II in CbpC was blocked by an additional loop that was absent in the other proteins (Fig. S4B), suggesting that CbpC might not be a functional sugar-binding protein. A Dali search using the CbpD structure revealed similarity with several monosaccharide-binding proteins in the closed conformation (Fig. S4D). A nearly closed substrate-binding pocket between domains I and II was found in the unliganded CbpD crystal structure, which is suitable for accommodating a monosaccharide (Fig. S4D). The small substrate-binding pocket suggests that CbpD cannot bind to cellodextrin, in agreement with our titration and genetic experimental results. CbpD has been proposed to be the transporter of xylose (36), but we could not detect any xylose-binding ability in either ITC or NMR experiments.

10.1128/mbio.01476-22.4 FIG S4 Structural analysis of CbpC and CbpD. (A) Overall structure of CbpC. The α-helices and β-strands are colored in green and orange, respectively. The hinge region includes three loops (colored blue), which connect the two domains. (B) Overlay of CbpC and the sugar-binding protein from Listeria innocua (PDB ID 5YSF, sophoropentaose complex). Inset shows that the substrate-binding site of CbpC is blocked by an internal loop (blue). (C) Overall structure of CbpD. The α-helices and β-strands are colored in pink and yellow, respectively. The hinge region includes three loops (colored blue), which connect the two domains. (D) Overlay of CbpD (pink) and the sugar-binding protein B1G1H7 (gray) from Burkholderia graminis (PDB ID 5HQJ, arabinose complex). Arabinose is shown as cyan sticks. Inset shows that CbpD has a similar substrate-binding pocket for an as-yet-unidentified monosaccharide. Download FIG S4, PDF file, 0.5 MB. Copyright © 2022 Yan et al. 2022 Yan et al. https://creativecommons.org/licenses/by/4.0/ This content is distributed under the terms of the Creative Commons Attribution 4.0 International license.

The structure of Lbp was determined to 1.50 Å resolution (Table S1, Fig. S5A). Although no sugar was added during crystallization, a clear electron density of a small molecular substrate was observed in the structure. However, neither laminaribiose nor cellobiose can fit into the density. Considering that Lbp has about 35% sequence identity with nucleoside-binding proteins (37, 38) as shown by a BLAST search, we tried to fit different nucleotides and found that guanosine fits well into the density (Fig. S5A). The guanosine has reasonable interactions with the surrounding residues (Fig. S5B), resembling the interactions of the nucleotide-binding protein from Treponema pallidum (PDB ID 2FQX) (Fig. S5C) which can bind various nucleotides (37). This suggests that Lbp may be a nucleotide-binding protein. Furthermore, the Δ*transporterL* strain grew well in the medium with laminaribiose as the sole carbon source (Fig. S5D). Further studies, however, are still required to elucidate the interaction of Lbp with laminaribiose and whether it can function in laminaribiose transport.

10.1128/mbio.01476-22.5 FIG S5 Structure of Lbp-guanosine. (A) Structure of Lbp in complex with guanosine. The α-helices and β-strands are colored in blue and yellow, respectively. The guanosine is shown as cyan sticks. In the inset, the 2mFo-DFc densities for guanosine are contoured in blue at 1.0 σ. (B) Guanosine-binding site of Lbp. Guanosine is shown as cyan sticks and residues from domains I and II are shown as blue and yellow sticks. Potential hydrogen bonds between guanosine and proteins are shown as dashed lines. (C) Comparison of the nucleotide-binding sites of Lbp (blue) and the nucleotide-binding protein PnrA (gray) from Treponema pallidum (PDB ID 2FQX, guanosine complex). (D) Growth curves of the Δ*transporterL* (red) and wild-type (black) strains on medium with laminaribiose as the sole carbon source. Download FIG S5, PDF file, 2.0 MB. Copyright © 2022 Yan et al. 2022 Yan et al. https://creativecommons.org/licenses/by/4.0/ This content is distributed under the terms of the Creative Commons Attribution 4.0 International license.

### Identification of the gene encoding the ATPase subunit of transporter B.

**FIG 6:**
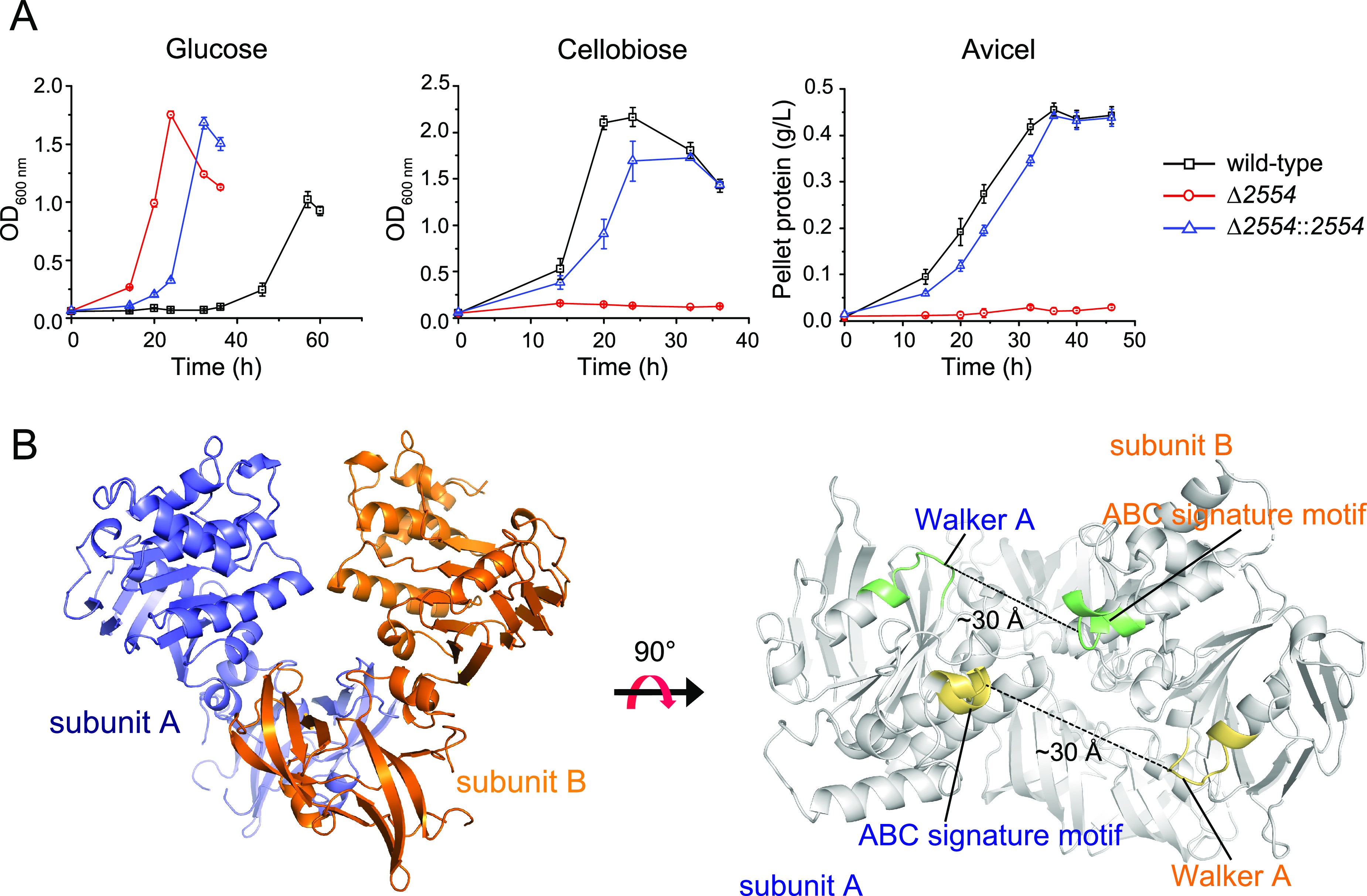
Function of Clo1313_2554 as the ATPase subunit of transporter B. (A) Growth curves of wild-type (black), *clo1313_2554*-deletion strain (red), and plasmid-based complementation strain (blue) on the designated carbon sources. OD_600_ and the pellet protein were measured on soluble (glucose and cellobiose) and insoluble (Avicel) carbon sources, respectively. All data represent the mean of triplicate cultures and the bars show ± SD. (B) Structural analysis of Clo1313_2554. Left panel shows the dimeric ATPase structure of Clo1313_2554; the two subunits are shown in blue and orange, respectively. Right panel shows that the two pairs of ATP binding motifs, i.e., the ABC signature motif and Walker A motif (green and orange, respectively), are separated by a distance of ~30 Å, indicating an open conformation.

As shown in a previous study (17), the gene cluster of transporter B in *C. thermocellum* lacks the gene encoding the nucleotide-binding domain NBD subunit (i.e., the ATPase subunit). Because *C. thermocellum* degrades cellulose into cellodextrins as the main form for uptake, the major cellodextrin transporter B is of major interest, and identification of its NBD gene is essential. Based on previously reported transcriptomic and proteomic studies (39–44), we noticed that one potential sugar-transporter ATPase gene, *clo1313_2554* in *C. thermocellum* DSM 1313 (*cthe_1862* in *C. thermocellum* ATCC 27405), was highly expressed together with transporter A and B genes. We therefore suspected that Clo1313_2554 represents the ATPase subunit of transporter B. Therefore, we deleted *clo1313_2554* using homologous recombination and obtained the Δ*2554* mutant. The Δ*2554* mutant lost the ability to grow on cellobiose and Avicel (Fig. 6A), in a manner similar to that of the Δ*transporterB* strain (Fig. 2). The growth phenotype was complemented successfully by expression of the *clo1313_2554* gene on a plasmid. In the genome, *clo1313_2554* is predicted to be a standalone gene, i.e., not part of any gene cluster (45). Therefore, the same phenotypes of the Δ*2554* and Δ*transporterB* mutants imply that Clo1313_2554 is the missing ATPase subunit of transporter B, which is the sole cellodextrin transporter in *C. thermocellum*.

To better confirm the function of Clo1313_2554, the crystal structure of Clo1313_2554 was determined at 2.8 Å resolution (Table S1). As shown in Fig. 6B, Clo1313_2554 presents the general α/β-type ATPase domain fold in homodimer form, similar to that of other NBD crystal structures (46–48). The ATP-binding Walker A motif from subunit A and the ABC signature motif from subunit B were approximately 30 Å apart in the structure, indicating that the nucleotide-free homodimer was in an open conformation (Fig. 6B).

### Inactivation of transporter B eliminated the inducer effect of cellobiose and Avicel for cellulosome expression.

**FIG 7:**
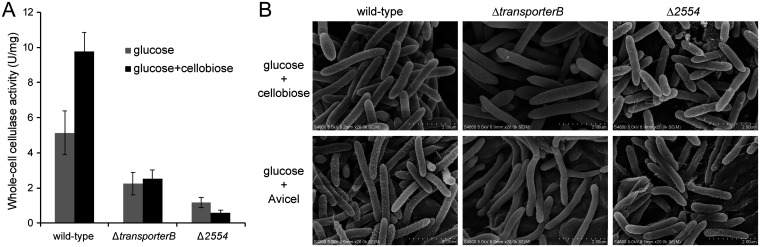
Cellulase activity (A) and scanning electron microscopic visualization (B) of wild-type, transporter B gene cluster deletion, and *clo1313_2554*-deletion strains of *C. thermocellum* grown on glucose, glucose supplemented with 5 g/L cellobiose, or glucose supplemented with 5 g/L Avicel.

Previous studies have shown that cellodextrin and Avicel can induce the expression of cellulosomes in *C. thermocellum* (49). Since transporter B is the sole cellodextrin transporter, we suspected that it may play a role in the induction of cellulosome expression. The cellulase activities of wild-type, Δ*transporterB*, and Δ*2554* cells were therefore compared in the glucose and glucose+cellobiose media. Wild-type cells exhibited a significant induction effect of cellobiose for cellulosome expression, while no induction effect was observed in Δ*transporterB* and Δ*2554* cells (Fig. 7A). In scanning electron microscope images of the cells, protuberance structures, indicative of cell surface-borne cellulosomes, can be clearly observed on wild-type cells cultured in glucose+cellobiose- or glucose+Avicel-containing media (Fig. 7B), indicating that the expression of poly cellulosomes is induced by cellobiose/Avicel (50). In contrast, very few protuberance-like structures were observed on the cell surfaces of Δ*transporterB* and Δ*2554* cells in media containing glucose supplemented with cellobiose or Avicel (Fig. 7B). These results indicate that cellodextrin transport and/or subsequent cellodextrin metabolism plays a key role in cellulosome production by the bacterium.

## DISCUSSION

Combined genetic, biophysical, and structural studies have provided solid evidence that transporter A and transporter B are the major glucose and cellodextrin transporters, respectively, among the five potential sugar transporters in *C. thermocellum*. These findings are quite unexpected, because many microorganisms are known to have a multiplicity of redundant sugar transporters for their major carbon source (51, 52). *C. thermocellum* takes up cellodextrins derived from cellulose as its major carbon source, while monosaccharides, such as glucose and fructose, can also be utilized by the bacterium after a lengthy adaptation period (21, 22). Although the physiological and evolutionary benefits of these restrictive transporters are still unknown, these findings suggest that engineering sugar transporters in *C. thermocellum* could be easier than in species with multiple redundant transporters.

The ATPase subunit of transporter B was identified as the encoded product of *clo1313_2554*, which is located outside the gene cluster of transporter B. Interestingly, this feature is found in many other bacteria. For example, the cellodextrin transporter gene cluster of Clostridium cellulolyticum H10 also lacks the ATPase gene, and Ccel_2909, which exhibits the highest homology to Clo1313_2554, was proposed to be its ATPase (53). This ATPase was demonstrated to be shared by several sugar transporters, and this phenomenon has been observed in several Gram-positive bacteria (53, 54). We noticed that both gene clusters of the β-glucosides transporter from the Gram-negative bacterium T. thermophilus HB8 and the Gram-positive bacterium Listeria innocua, which include a CbpB homologous gene, also lack a gene for the ATPase subunit (35). Therefore, the non-cluster location of *clo1313_2554* appears to be a conserved feature in some transporters in both Gram-negative and Gram-positive bacteria. Although this feature was proposed to be the basis for shared ATPase by several sugar transporters (53, 54), the potential benefit of this arrangement in transporter B of *C. thermocellum* remains to be elucidated.

Contrary to previous reports (17), transporter A in *C. thermocellum* was confirmed to be the sole glucose transporter in our study, instead of transporter C. This finding is indeed consistent with those of two recent studies involving the adaptive evolution of *C. thermocellum* on glucose and fructose, which showed that transporter A genes contained the most frequent mutations in the adaptive strains (21, 22). Unlike other monosaccharide-binding proteins with strong affinities to the substrate (*K*_D_ in the μM range) (51), the binding of CbpA to glucose is remarkably weak as shown by ITC experiments. This weak binding might account for the impaired growth on glucose displayed by *C. thermocellum*. The weak ability of glucose uptake is consistent with the sugar metabolism of *C. thermocellum* in which cellodextrins metabolized through a phosphorolytic mechanism have more bioenergetic benefits than glucose (55).

At this stage, we have no satisfactory explanation for the contradictory results in the binding properties of CbpC and CbpD compared to the previous study by Nataf et al. (17). However, regardless of the exact specificity of CbpC and CbpD, the knockout mutants of these associated transporters clearly indicate that they do not play a significant role in the utilization of cellulose by *C. thermocellum.*

The data in this study showed that transporter B is related to cellulosome expression induced by cellodextrins and Avicel. Although in lignocellulolytic fungi, various sugar transporters are closely related to cellulase induction and production (52, 56), the relationship in bacterial systems is currently unclear. Since bacterial sugar transporters and the cellulosome system components differ from fungal transporters and cellulases, future studies are needed to address the regulation mechanism of transporter B inactivation on cellulosome expression in *C. thermocellum*. Recent studies have shown that some solute-binding proteins of bacterial ABC transporters, including the maltose- and xylose-binding proteins, can activate bacterial receptors for signal transduction with diverse mechanisms (57). Previous studies have shown that several distinct sigma/anti-sigma factors (SigI/RsgI) are responsible for substrate sensing (i.e., RsgI is the receptor) and cellulosome expression (58–61). Therefore, future studies should focus on whether and how transporter B is coupled to SigI/RsgI factors in the regulation of cellulosome expression. These studies will provide both mechanistic insights into cellulosome production and knowledge for effective rational engineering of *C. thermocellum* for lignocellulose biorefineries.

## MATERIALS AND METHODS

### Bacterial strains and cultivation.

The bacterial strains used in this study are listed in Table S2 in the supplemental material. *C. thermocellum* cells were grown anaerobically at 60°C in GS-2 medium with 5 g/L glucose, cellobiose, laminaribiose, or Avicel as the sole carbon source (62). When necessary, the media were supplemented with 3 μg/mL thiamphenicol (Tm), 10 μg/mL 5-fluoro-2-deoxyuridine (FUDR), and 500 μg/mL 5-fluoroorotic acid (FOA). Cell growth of the *C. thermocellum* strains on glucose, cellobiose, or laminaribiose was determined by monitoring the optical density at 600 nm (OD_600_) every 4 to 8 h. To determine cell growth on Avicel, the cell pellet proteins were quantified by the Bradford method as previously described (24).

10.1128/mbio.01476-22.7 TABLE S2 Bacterial strains and plasmids used in this study. Download Table S2, PDF file, 0.1 MB. Copyright © 2022 Yan et al. 2022 Yan et al. https://creativecommons.org/licenses/by/4.0/ This content is distributed under the terms of the Creative Commons Attribution 4.0 International license.

### Plasmid construction.

All plasmids for gene expression, inactivation, or deletion were constructed using the E. coli Top10 strain and verified by colony PCR and sequencing using the primers listed in Table S3. The thermotargetrons for gene inactivation in *C. thermocellum* were constructed with pHK-TT1A as the template as previously described (23). The resulting targetrons are denoted by a number which corresponds to the 5′ nucleotide residue of the targetron insertion site within the target gene, followed by an “a” or “s” indicating the antisense/bottom or sense/top strand of the insertion site (Table S3). To construct plasmids for gene deletion via homologous recombination in *C. thermocellum*, upstream and downstream homology regions of ~1.2 Kb were obtained by PCR with the genomic DNA of *C. thermocellum* DSM1313 as the template and corresponding primers (Table S3) and were ligated to plasmid pHK-HR as previously described (25). For gene complementation in *C. thermocellum*, the target genes were successively cloned into pHK-*P_2638_*-BGL to replace the *bgl* gene using a seamless cloning kit (Vazyme Biotech, Beijing, China). Gene expression would be driven by the promoter of gene *clo1313_2638* as previously reported (63). All obtained plasmids for gene inactivation, deletion, and complementation were verified using the primer sets TT1A-F/R, HR-F/R, and pHK-F/R, respectively.

10.1128/mbio.01476-22.8 TABLE S3 Primers used in this study. Underlined sequences indicate the restriction sites. Download Table S3, PDF file, 0.1 MB. Copyright © 2022 Yan et al. 2022 Yan et al. https://creativecommons.org/licenses/by/4.0/ This content is distributed under the terms of the Creative Commons Attribution 4.0 International license.

### Electrotransformation and screening of *C. thermocellum*.

The plasmids constructed in E. coli Top10 were transformed into E. coli BL21(DE3) to remove Dcm methylation. Plasmid transformation to *C. thermocellum* DSM1313 was then performed via electroporation using 0.1-cm electroporation cuvettes and a homemade electroporator with a pulse amplitude of 1.5 kV as previously described (24). After electroporation, cells were recovered for 12 h at 51°C in 4 mL of GS-2 medium before screening on a solid medium containing Tm and 5 g/L cellobiose or glucose as the sole carbon source. The obtained colonies were screened by colony PCR and sequencing as the target transformants.

Transformants containing the thermotargetron plasmid were analyzed by colony PCR using the primer set TT1A-F/R. The precise targetron insertion in each of the target genes in the genome DNA was then determined using the corresponding sequencing primers (Table S3). After gene targeting, the plasmids were cured by continuous inoculation and growth of cells in a Tm-free fresh medium (23). Transformants containing homologous recombination plasmid were screened following a previously described two-step protocol (25). The seamless deletion of the operons of transporters A and B and the gene *clo1313_2554* was subsequently verified by colony PCR and sequencing using the primer sets ΔA-F/R, ΔB-F/R, and Δ2554-F/R, respectively.

### Protein expression and purification.

To facilitate heterologous expression and crystallization, the genes encoding CbpA to CbpD, Lbp, and Clo1313_2554 proteins without signal peptide were amplified with primers shown in Table S3 using the genome of *C. thermocellum* DSM1313 as a template. The DNA fragments were digested by the BamHI and XhoI restriction enzymes and then ligated into the pET28a-SMT3 vector. The encoded target proteins in these expression vectors contain an N-terminal His_6_-SMT3 tag, which could be removed by ULP1 protease treatment in the purification procedures. After sequencing, the expression plasmids were transformed into E. coli BL21(DE3) to produce the target proteins.

The proteins were purified by affinity chromatography, ULP1 protease treatment, and gel filtration as previously described (64), except that the final buffer for gel filtration was 10 mM Tris-HCl and 100 mM NaCl (pH 8.0). Protein concentration was determined by UV absorption at 280 nm using the theoretical molar extinction coefficient.

Selenomethionine derivatives of CbpB and CbpC were expressed in an M9 minimal medium supplemented with selenomethionine, lysine, valine, threonine, leucine, isoleucine, and phenylalanine. ^15^N-labeled proteins for NMR experiments were obtained by cell cultivation using M9 minimal medium containing ^15^N-NH_4_Cl as the sole nitrogen source. The labeled proteins were purified using the same procedures as for the unlabeled proteins.

### Isothermal titration calorimetry measurements.

Isothermal titration calorimetry measurements were performed at 30°C using a MicroCal PEAQ-ITC (Malvern Panalytical Ltd., Malvern, United Kingdom). Protein samples were dialyzed against buffer containing 50 mM Tris-HCl (pH 8.0) and 100 mM NaCl. Carbohydrate-ligand solutions of cellodextrins, glucose, and xylose were prepared using the same buffer as for protein dialysis. The sample cell was loaded with 280 μL of protein sample (~50 μM), and the reference cell contained distilled water. The syringe was filled with 50 μL of ligand (500 to 750 μM). Titrations were carried out by adding 0.8 μL of ligand for the first injection and 2 μL for the subsequent 19 injections, with stirring at 750 rpm. Binding parameters were determined by fitting the experimental binding isotherms using a single-site model.

### NMR titration.

NMR titration experiments were performed at 298 K on a Bruker Avance III 600 MHz NMR spectrometer equipped with a cryoprobe. The ^15^N-labeled protein samples contained 0.1 or 0.2 mM sugar-binding protein in 20 mM Bis-Tris (pH 6.5), 100 mM KCl, 0.02% (wt/vol) dextran sulfate sodium, and 90% H_2_O/10% D_2_O. The concentrations of carbohydrate substrates were 10, 20, or 100 mM in the same buffer. For the titration, the substrates were gradually added to the labeled proteins until the ratio of their concentrations reached 1:20. A series of two-dimensional ^1^H-^15^N HSQC spectra were recorded at different protein-substrate ratios during the titration.

### Crystallization, data collection, structure determination, and refinement.

The purified proteins for crystallization were concentrated to approximately 20 mg/mL in 10 mM Tris-HCl (pH 8.0) and 100 mM NaCl. Crystals were obtained using sitting-drop vapor diffusion for screening and hanging-drop vapor diffusion for optimization at 18°C. To obtain crystals of the Sbp/carbohydrate complex, CbpA protein was mixed with glucose at a 1:50 molar ratio, while CbpB was mixed with each cellodextrin (G2 to G5) at a 1:5 molar ratio. High-quality crystals were obtained under the conditions shown in Table S1. All of the crystals used for data collection were cryoprotected by soaking in well solution supplemented with 20% (vol/vol) glycerol for 10 s, and then flash-cooled in liquid nitrogen. X-ray diffraction data were collected on the BL17U1 or BL19U1 beamline at the Shanghai Synchrotron Radiation Facility (65–67).

The diffraction data indexing, integration, and scaling were conducted using XDS (68). The crystal structures of CbpA, CbpD, Lbp, and Clo1313_2554 were determined by molecular replacement using the protein tmGBP (PDB ID 2H3H) (30), XylFII (PDB ID 5XSS) (31), PnrA (PDB ID 2FQX) (37), and an NBD from Pyrococcus horikoshii (PDB ID 1V43) (69), respectively, as search models in the PHENIX program Phaser-MR (70, 71). The structures of the CbpB/cellotriose complex and CbpC Se-derivative were determined by single-wavelength anomalous dispersion phasing using CCP4 CRANK2 (72, 73). One monomer from the resulting structure was subsequently used as the search model for molecular replacement to determine the structures of ligand-free CbpB, CbpC, and the CbpB-cellodextrin complexes. Refinements of the structures were performed using Coot (74) and PHENIX (71). Carbohydrates in all structures were further validated using Privateer in CCP4 (72, 75). All structure figures were made using PyMOL (Schrödinger LLC).

### Scanning electron microscopy.

Scanning electron microscopy was performed with *C. thermocellum* cells according to a previously reported method using a field emission scanning electron microscope S-4800 (Hitachi, Tokyo, Japan) (24).

### Cellulosome activity measurement.

*C. thermocellum* cells were cultivated to the early exponential stage with various carbon sources. Cell suspensions (1.0 mL) were sampled and centrifuged at 15,000 × *g* for 10 min to separate supernatant from the pellet. Cell-associated cellulosome activity was measured in a 1.0-mL reaction volume containing the pellet and 15 mg Avicel as the substrate, using GS-2 medium as the reaction buffer. The concentration of reducing sugars was determined by the 3,5-dinitrosalicylic acid method after incubation at 55°C for 24 h unless otherwise stated. One unit of enzyme activity is defined as the amount of enzyme that releases 1 nmol reducing sugar (glucose equivalent) per min.

### Data availability.

Atomic coordinates and structure factors have been deposited in the PDB. The accession numbers are listed in Table S1.
