# Supplementary figures and images for: Deciphering Cellodextrin and Glucose Uptake in Clostridium thermocellum
Source: mBio. 2022 Sep 7;13(5):e01476-22. doi: 10.1128/mbio.01476-22 (PMC9601137; doi:10.1128/mbio.01476-22)

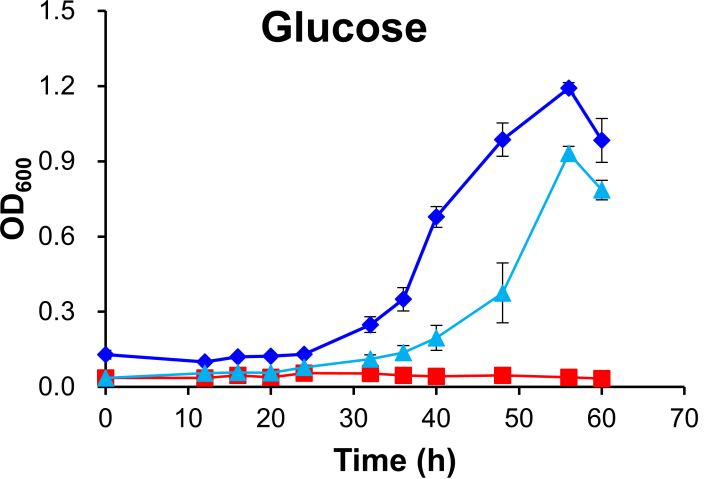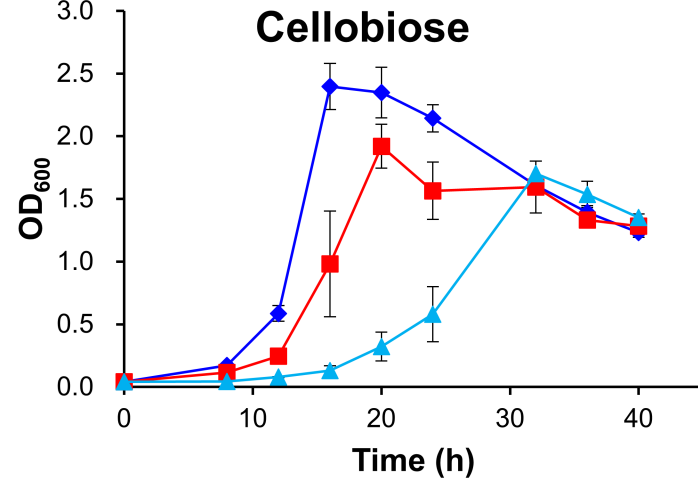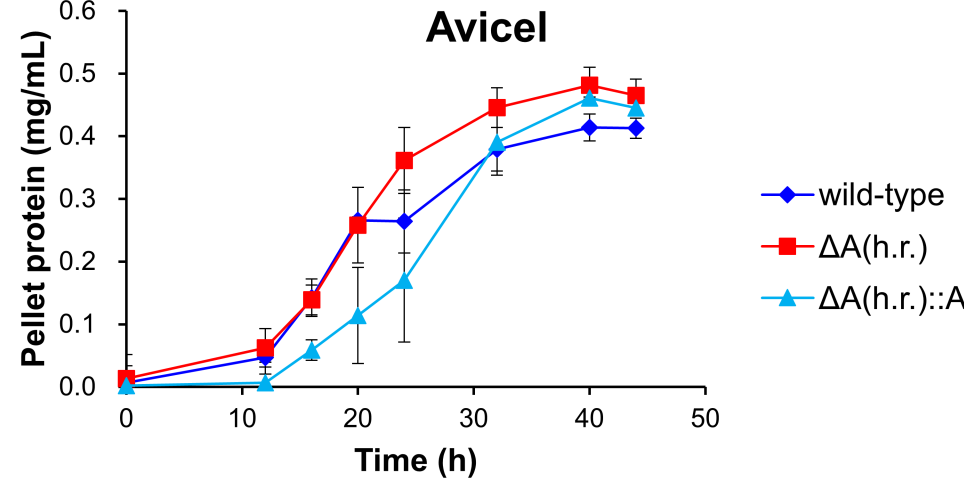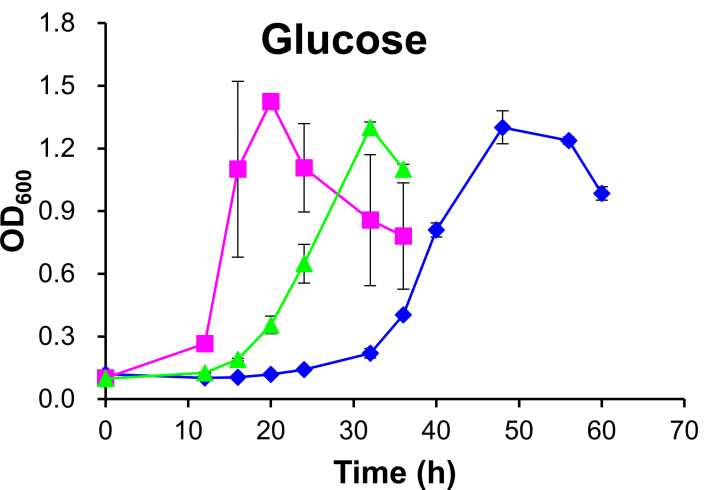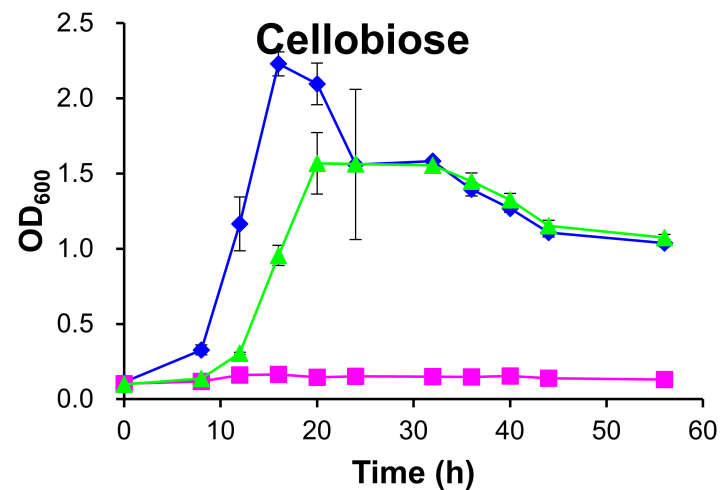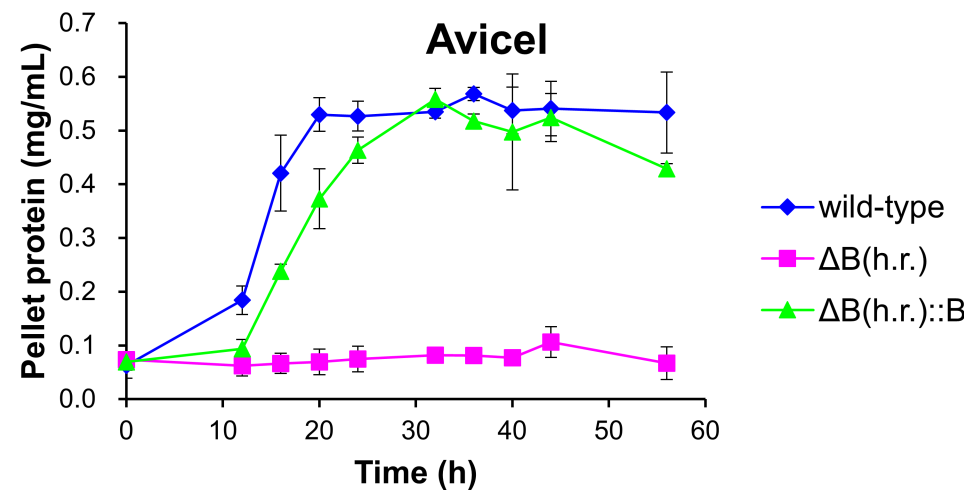

Supplement: FIG S1 [file mbio.01476-22-s0001.pdf]

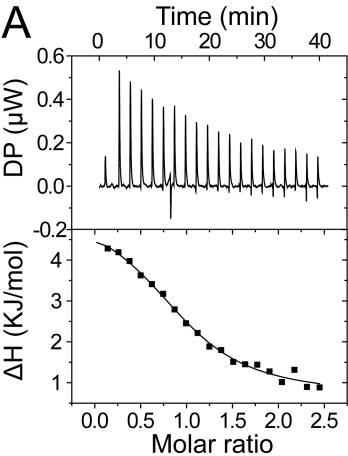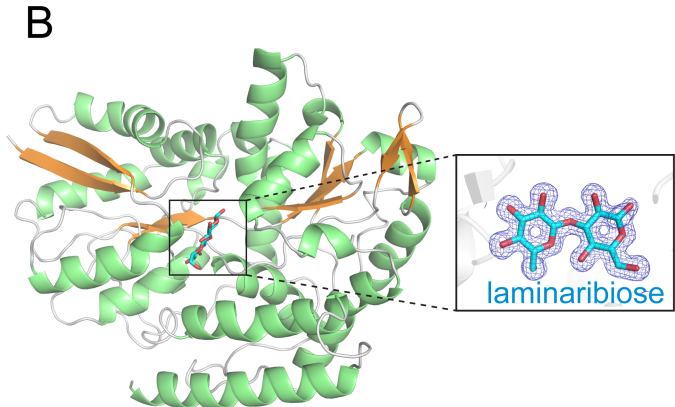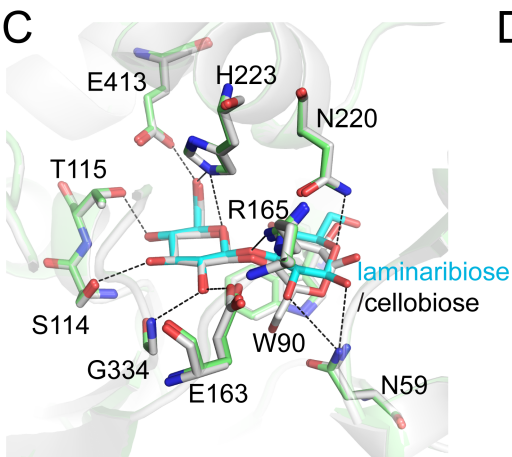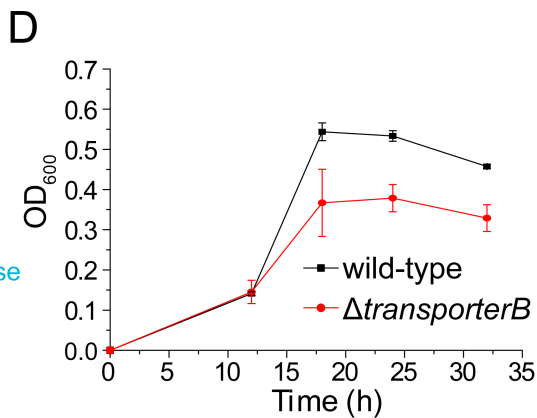

Supplement: FIG S3 [file mbio.01476-22-s0003.pdf]

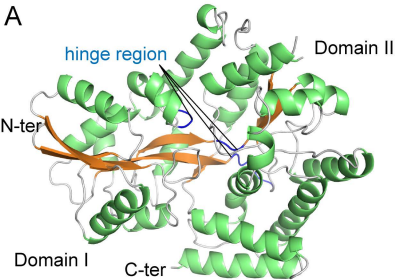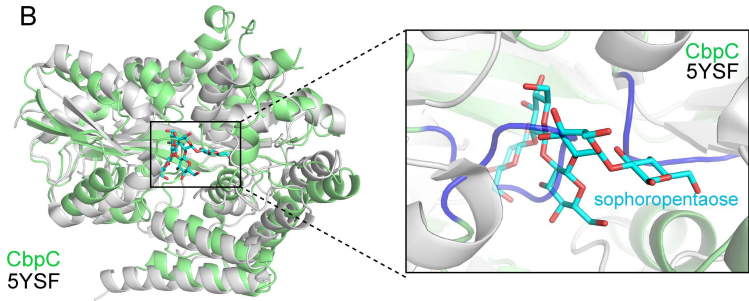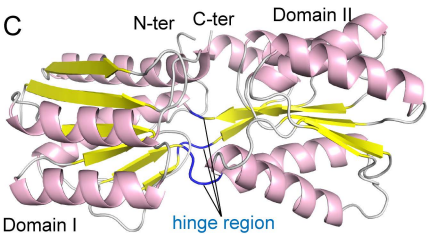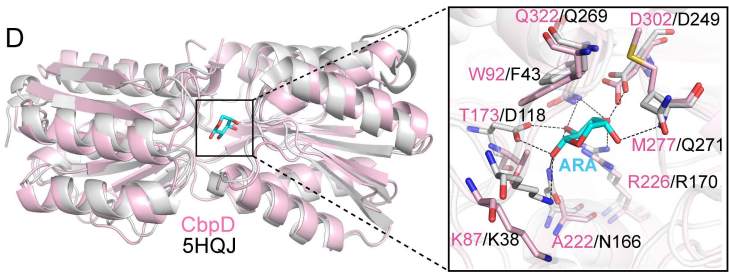

Supplement: FIG S4 [file mbio.01476-22-s0004.pdf]

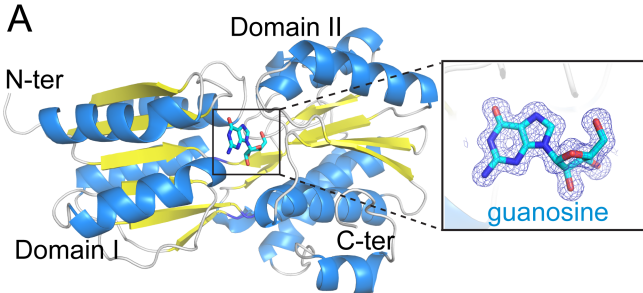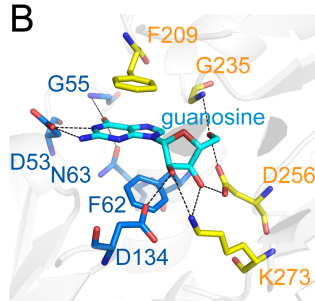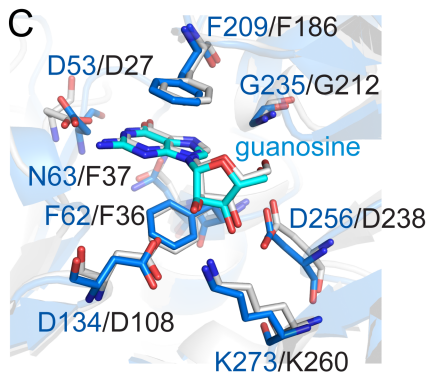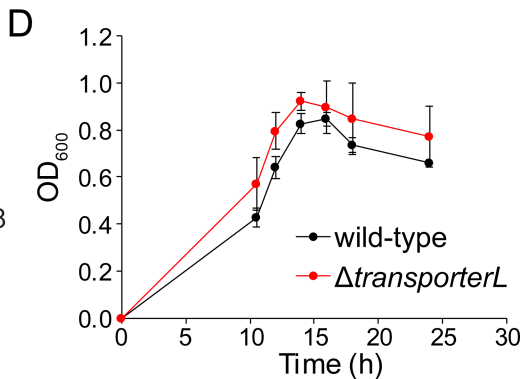

Supplement: FIG S5 [file mbio.01476-22-s0005.pdf]
