# Supplementary material for: Deciphering Cellodextrin and Glucose Uptake in Clostridium thermocellum
Source: mBio. 2022 Sep 7;13(5):e01476-22. doi: 10.1128/mbio.01476-22 (PMC9601137; doi:10.1128/mbio.01476-22)
Supplement: FIG S2 [file mbio.01476-22-s0002.pdf]

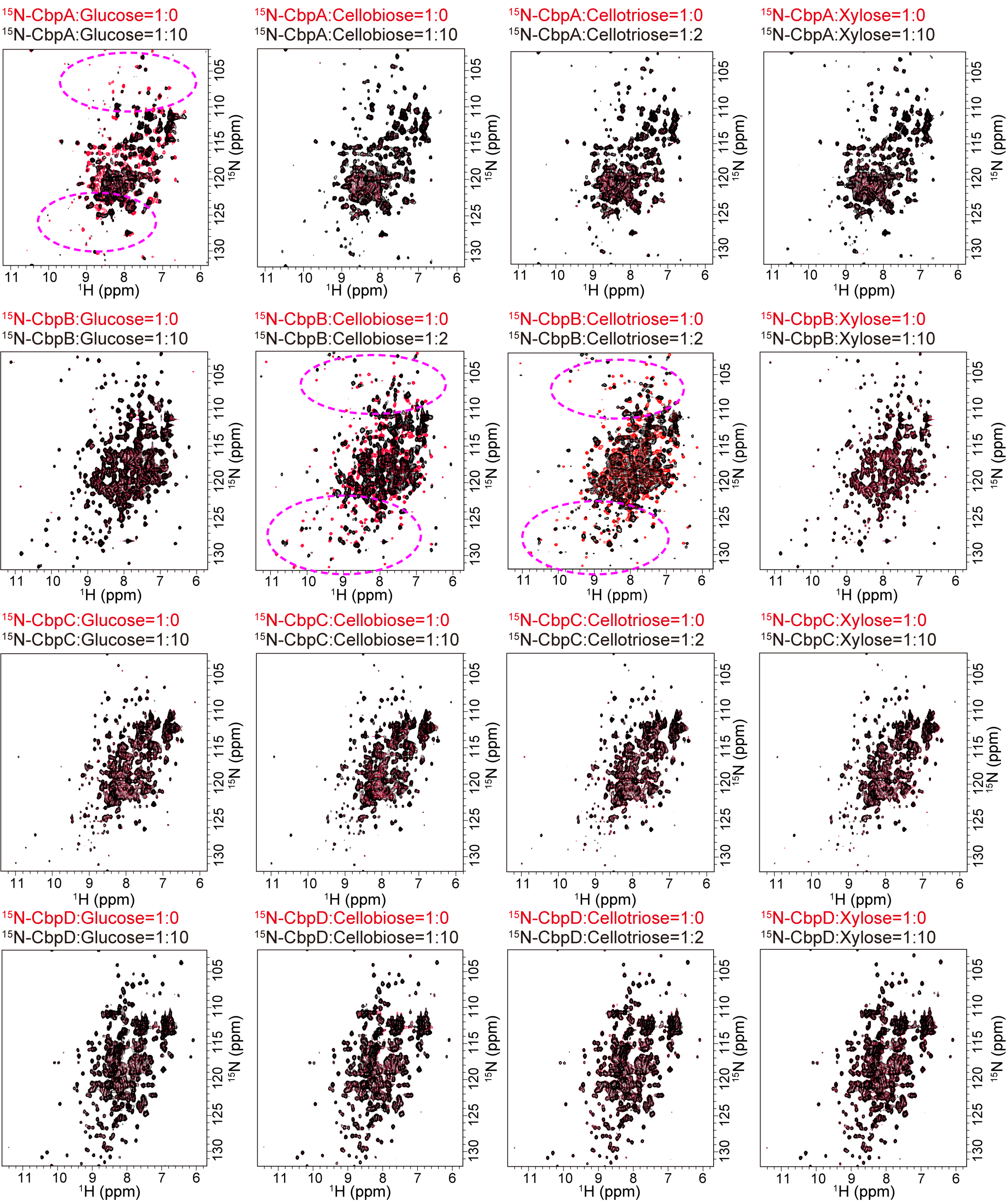
^{15}N-CbpA:Glucose=1:0
^{15}N-CbpA:Glucose=1:10
^{15}N-CbpA:Cellobiose=1:0
^{15}N-CbpA:Cellobiose=1:10
^{15}N-CbpA:Cellotriose=1:0
^{15}N-CbpA:Cellotriose=1:2
^{15}N-CbpA:Xylose=1:0
^{15}N-CbpA:Xylose=1:10
^{15}N-CbpB:Glucose=1:0
^{15}N-CbpB:Glucose=1:10
^{15}N-CbpB:Cellobiose=1:0
^{15}N-CbpB:Cellobiose=1:2
^{15}N-CbpB:Cellotriose=1:0
^{15}N-CbpB:Cellotriose=1:2
^{15}N-CbpB:Xylose=1:0
^{15}N-CbpB:Xylose=1:10
^{15}N-CbpC:Glucose=1:0
^{15}N-CbpC:Glucose=1:10
^{15}N-CbpC:Cellobiose=1:0
^{15}N-CbpC:Cellobiose=1:10
^{15}N-CbpC:Cellotriose=1:0
^{15}N-CbpC:Cellotriose=1:2
^{15}N-CbpC:Xylose=1:0
^{15}N-CbpC:Xylose=1:10
^{15}N-CbpD:Glucose=1:0
^{15}N-CbpD:Glucose=1:10
^{15}N-CbpD:Cellobiose=1:0
^{15}N-CbpD:Cellobiose=1:10
^{15}N-CbpD:Cellotriose=1:0
^{15}N-CbpD:Cellotriose=1:2
^{15}N-CbpD:Xylose=1:0
^{15}N-CbpD:Xylose=1:10
^{1}H (ppm)
^{15}N (ppm)
11 10 9 8 7 6
105 110 115 120 125 130
