# Supplementary material for: Deciphering Cellodextrin and Glucose Uptake in Clostridium thermocellum
Source: mBio. 2022 Sep 7;13(5):e01476-22. doi: 10.1128/mbio.01476-22 (PMC9601137; doi:10.1128/mbio.01476-22)
Supplement: TABLE S1 [file mbio.01476-22-s0006.pdf]

Table S1. Crystallographic data collection and refinement statistics.

Parameter	CbpA	CbpA+Glc	CbpB
**PDB code**	7X0G	7X0H	7X0I
**Crystallization**	0.2 M $MgCl_2$, 0.1 M Tris, pH8.5, 23% w/v Polyethylene glycol 3350	0.1 M HEPES, pH 7.5, 2.0 M Ammonium sulfate	0.1 M Sodium acetate, pH 4.5, 18% w/v Polyethylene glycol 3350
**Data collection**[a]			
Space group	C 1 2 1	C 2 2 21	P 21 21 21
a,b,c (Å)<br>*α, β, γ* (°)	124.07, 94.46, 58.50<br>90.00, 114.94, 90.00	75.87, 234.62, 196.32<br>90.00,90.00, 90.00	63.46, 67.53, 180.55<br>90.00, 90.00, 90.00
Wavelength (Å)	0.979	0.979	0.979
Resolution (Å)	50-2.10 (2.15-2.10)	50.00-1.85 (1.90-1.85)	50.00-1.70 (1.74-1.70)
Unique reflections	68775 (5175)	288959 (21477)	163957 (12167)
Completeness (%)	97.5 (99.0)	100(100)	99.6 (99.6)
Redundancy	1.9 (1.9)	6.9 (7.0)	6.8 (6.7)
Mean I/sigma (I)	6.08 (2.98)	9.56 (2.00)	12.41 (2.09)
R_{merge}[b]	0.09 (0.22)	0.16 (1.00)	0.12 (0.99)
**Refinement**			
R_{work}/R_{free} (%)	26.14/28.64	16.19/18.82	16.51/20.22
**No. atoms**			
Protein	4234	8512	6333
Ligand	0	48	0
water	298	1531	732
**B-factors**			
Average B-factor	56.10	22.40	29.24
Proteins	56.89	20.30	28.39
Ligand	-	13.21	-
Solvent	44.88	34.33	36.52
**r.m.s.d.**			
Bond length (Å)	0.006	0.006	0.008
Bond angles (°)	1.06	1.19	1.15
**Ramachandran statistics**			
Favored (%)	95.27	97.89	98.65
Outliers (%)	0.00	0.00	0.00

a. Values in parentheses refer to data in the highest-resolution shell.
b. $R_{merge}=\Sigma_{hkl}\Sigma_i|I(hkl)_i - <I(hkl)>|/\Sigma_{hkl}\Sigma_i <I(hkl)_i>$, where *I* is the observed intensity, $<I(hkl)>$ represents the average intensity, and $I(hkl)_i$ represents the observed intensity of each unique reflection.

Table S1. (continued)

**Parameter**	**CbpB-G2**	**SeMet-CbpB-G3**	**CbpB-G4**
**PDB code**	7X0J	7X0K	7X0L
**Crystallization**	0.1 M Sodium acetate, pH 4.5, 18% w/v Polyethylene glycol 3350	0.2 M Ammonium phosphate monobasic, pH 4.6, 22% w/v Polyethylene glycol 3350	0.1M Bis-Tris, pH5.5, 25% w/v Polyethylene glycol 3350
**Data collection**[a]			
Space group	P 21 21 21	P 1 21 1	P 1 21 1
a,b,c (Å)<br>*α, β, γ* (°)	45.95, 72.82, 230.60<br>90.00, 90.00, 90.00	69.44, 44.15, 113.06<br>90.00, 103.98, 90.00	72.07, 46.56, 119.10<br>90.00, 107.87, 90.00
Wavelength (Å)	0.979	0.979	0.979
Resolution (Å)	50.00-1.70 (1.74-1.70)	50.00-1.70 (1.74-1.70)	50-1.90 (1.95-1.90)
Unique reflections	162939 (12088)	136987 (10092)	112089 (7619)
Completeness (%)	99.1 (99.9)	95.6 (95.8)	96.4 (88.0)
Redundancy	3.4 (3.4)	3.5 (3.5)	3.1 (2.9)
Mean I/sigma (I)	5.82 (1.65)	6.88 (1.54)	8.15 (2.45)
R_{merge}[b]	0.13 (0.71)	0.11 (0.81)	0.15 (0.98)
**Refinement**			
R_{work}/R_{free} (%)	17.98/21.12	17.96/21.56	17.44/20.71
**No. atoms**			
Protein	6297	6298	6342
Ligand	46	68	90
water	816	943	579
**B-factors**			
Average B-factor	28.74	20.29	24.04
Proteins	27.68	18.97	23.37
Ligand	16.56	17.33	24.53
Solvent	37.56	29.32	31.40
**r.m.s.d.**			
Bond length (Å)	0.005	0.005	0.006
Bond angles (°)	1.03	1.00	1.06
**Ramachandran statistics**			
Favored (%)	98.39	98.52	98.28
Outliers (%)	0.00	0.00	0.00

a. Values in parentheses refer to data in the highest-resolution shell.

b. $R_{merge}=\Sigma_{hkl}\Sigma_i|I(hkl)_i-\langle I(hkl)\rangle|/\Sigma_{hkl}\Sigma_i\langle I(hkl)_i\rangle$, where I is the observed intensity, $\langle I(hkl)\rangle$ represents the average intensity, and $I(hkl)_i$ represents the observed intensity of each unique reflection.

Table S1. (continued)

**Parameter**	**CbpB+G5**	**CbpB+L2**	**NbdB (Clo1313_2554)**
**PDB code**	7X0M	7X0N	7X0Q
**Crystallization**	0.1 M Sodium acetate, pH 4.5, 20% w/v Polyethylene glycol 3350	2% Tacsimate pH 4.0, 0.1 M Sodium acetate trihydrate pH 4.6, 16% w/v polyethylene glycol 3350	0.1 M Acetate/acetic acid pH4.4, 0.2 M Lithium sulfate, 2 M Sodium chloride
**Data collection**[a]			
Space group	P 1 21 1	P 1 21 1	P 43 21 2
a,b,c (Å)<br>*α, β, γ* (°)	72.32, 46.51, 119.38<br>90.00, 107.61, 90.00	72.75, 46.44, 116.49<br>90.00, 106.58, 90.00	104.39, 104.39, 167.78<br>90.00, 90.00, 90.00
Wavelength (Å)	0.979	0.979	0.979
Resolution (Å)	50.00-2.00 (2.05-2.00)	40.00-1.68 (1.72-1.68)	36.91-2.90 (2.98-2.90)
Unique reflections	83500 (6481)	148751 (11347)	39211 (2859)
Completeness (%)	83.3 (87.8)	89.2 (91.3)	100 (100)
Redundancy	1.9 (2.0)	1.9 (1.9)	6.8 (7.1)
Mean I/sigma (I)	7.12 (3.98)	11.3 (2.4)	16.7 (2.8)
R_{merge}[b]	0.12 (0.47)	0.05 (0.43)	0.07 (0.66)
**Refinement**			
R_{work}/R_{free} (%)	17.90/20.88	17.34/20.99	22.84/27.59
**No. atoms**			
Protein	6294	6317	5594
Ligand	112	46	0
water	594	804	0
**B-factors**			
Average B-factor	22.60	26.95	101.49
Proteins	21.61	26.21	101.49
Ligand	33.40	15.25	-
Solvent	31.01	33.40	-
**r.m.s.d.**			
Bond length (Å)	0.006	0.005	0.007
Bond angles (°)	1.11	1.10	1.25
**Ramachandran statistics**			
Favored (%)	99.01	99.02	94.50
Outliers (%)	0.00	0.00	0.00

a. Values in parentheses refer to data in the highest-resolution shell.

b. $R_{merge}=\Sigma_{hkl}\Sigma_i|I(hkl)_i-\langle I(hkl)\rangle|/\Sigma_{hkl}\Sigma_i\langle I(hkl)_i\rangle$, where *I* is the observed intensity, $\langle I(hkl)\rangle$ represents the average intensity, and $I(hkl)_i$ represents the observed intensity of each unique reflection.

Table S1. (continued)

**Parameter**	**SeMet-CbpC**	**CbpC**	**CbpD**
**PDB code**	-	7X0O	7X0P
**Crystallization**	0.2 M Sodium malonate pH 5.0, 20% w/v Polyethylene glycol 3350	0.1M Bis-Tris pH 5.5, 25% w/v Polyethylene glycol 3350	0.1 M Sodium acetate, pH 4.5, 20% w/v Polyethylene glycol 3350
**Data collection**[a]			
Space group	P 21 21 21	P 21 21 21	C 1 2 1
a,b,c (Å)<br>*α, β, γ* (°)	40.09, 83.34, 119.72<br>90.00, 90.00, 90.00	67.81, 80.06, 170.35<br>90.00, 90.00, 90.00	69.91, 63.17, 69.70<br>90.00, 106.20, 90.00
Wavelength (Å)	0.979	0.979	0.979
Resolution (Å)	50.00-2.70 (2.77-2.70)	50.00-2.00 (2.05-2.00)	50-1.50 (1.54-1.50)
Unique reflections	21133 (1534)	103492 (5606)	86939 (6443)
Completeness (%)	99.8 (99.4)	85.6 (62.7)	94.7 (95.2)
Redundancy	3.4 (3.5)	3.5 (2.5)	1.9 (1.9)
Mean I/sigma (I)	11.4 (1.92)	9.50 (1.47)	10.31 (1.91)
R_{merge}[b]	0.07(0.82)	0.09 (0.69)	0.04 (0.51)
**Refinement**			
R_{work}/R_{free} (%)		23.13/25.97	16.98/19.99
**No. atoms**			
Protein		6637	2341
Ligand		0	0
water		397	363
**B-factors**			
Average B-factor		41.12	33.88
Proteins		41.18	32.87
Ligand		-	-
Solvent		40.10	40.40
**r.m.s.d.**			
Bond length (Å)		0.004	0.010
Bond angles (°)		1.00	1.29
**Ramachandran statistics**			
Favored (%)		96.86	98.71
Outliers (%)		0.00	0.00

a. Values in parentheses refer to data in the highest-resolution shell.
b. $R_{merge}=\Sigma_{hkl}\Sigma_i|I(hkl)_i-\langle I(hkl)\rangle|/\Sigma_{hkl}\Sigma_i\langle I(hkl)_i\rangle$, where I is the observed intensity, $\langle I(hkl)\rangle$ represents the average intensity, and $I(hkl)_i$ represents the observed intensity of each unique reflection.

Table S1. (continued)

**Parameter**	**Lbp+ guanosine**
**PDB code**	7X0R
**Crystallization**	0.2 M Zinc acetate, 16% w/v Polyethylene glycol 3350
**Data collection**[a]	
Space group	P 1 21 1
a,b,c (Å)<br>*α, β, γ* (°)	53.83, 62.09, 81.30<br>90.00, 101.70, 90.00
Wavelength (Å)	0.979
Resolution (Å)	40.00-1.47(1.51-1.47)
Unique reflections	165622 (11830)
Completeness (%)	94.4 (91.3)
Redundancy	1.8 (1.6)
Mean I/sigma (I)	9.7 (2.2)
R_{merge}[b]	0.05 (0.37)
**Refinement**	
R_{work}/R_{free} (%)	15.65/17.36
**No. atoms**	
Protein	4629
Ligand	48
water	533
**B-factors**	
Average B-factor	19.83
Proteins	18.41
Ligand	14.02
Solvent	32.73
**r.m.s.d.**	
Bond length (Å)	0.008
Bond angles (°)	1.29
**Ramachandran statistics**	
Favored (%)	97.58
Outliers (%)	0.00

a. Values in parentheses refer to data in the highest-resolution shell.
b. $R_{merge}=\Sigma_{hkl}\Sigma_i|I(hkl)_i-<I(hkl)>|/\Sigma_{hkl}\Sigma_i<I(hkl)_i>$, where I is the observed intensity, $<I(hkl)>$ represents the average intensity, and $I(hkl)_i$ represents the observed intensity of each unique reflection.
