# Supplementary material for: Deciphering Cellodextrin and Glucose Uptake in Clostridium thermocellum
Source: mBio. 2022 Sep 7;13(5):e01476-22. doi: 10.1128/mbio.01476-22 (PMC9601137; doi:10.1128/mbio.01476-22)
Supplement: TABLE S2 [file mbio.01476-22-s0007.pdf]

Table S2. Bacterial strains and plasmids used in this study.

**Strains/plasmids**	Relevant characteristic	Sources
**Strains**		
E. coli		
Top10	*F- mcrA Δ(mrr-hsdRMS-mcrBC) F80lacZΔM15 ΔlacX74 nupG recA1 araD139 Δ(ara-leu)7697 galE15 galK16 rpsL(StrR) endA1 λ-*	Transgen Biotech Co.
BL21(DE3)	F^-*, ompT, gal, dcm, lon,* $hsdS_B(r_B^- m_B^-)$ λ (DE3 [*lacI* lacUV5-T7 gene 1 *ind1 sam7 nin5*])	Transgen Biotech Co.
C. thermocellum		
DSM1313	Wild type stain	DSMZ
Δ*transporterA*	Derived from DSM1313, *transporterA* gene inactivated	This work
Δ*transporterB*	Derived from DSM1313, *transporterB* gene inactivated	This work
Δ*transporterC*	Derived from DSM1313, *transporterC* gene inactivated	This work
Δ*transporterD*	Derived from DSM1313, *transporterD* gene inactivated	This work
Δ*transporterL*	Derived from DSM1313, *transporterL* gene inactivated	This work
Δ*pyrF*	Derived from DSM1313, *pyrF* gene deletion	(1)
Δ*pyrF*Δ*transporterA*	Derived from Δ*pyrF*, *transporterA* gene deletion	This work
Δ*pyrF*Δ*transporterB*	Derived from Δ*pyrF*, *transporterB* gene deletion	This work
Δ*pyrF*Δ*2554*	Derived from Δ*pyrF*,*Clo1313_2554* gene deletion	This work
Δ*transporterA*:: pHK-*transporter A*	Derived from Δ *transporterA*, with the plasmid pHK-*transporterA*	This work
Δ*transporterB*:: pHK-*transporter B*	Derived from Δ*transporterB* with the plasmid pHK-*transporterB*	This work
Δ*2554*:: pHK-*2554*	Derived from Δ*2554*, with the plasmid pHK-2554	This work
**Plasmids**		
pHK	pNW33N derivative, *E. coli*-*C. thermocellum* shuttle vector, CmR/TmR	(1)
pHK-P_{2638}-BGL	pHK derivative, containing BGL expression cassette using the clo1313_2638 promoter	(2)
pHK-*transporterA*	pHK-P_{2638}-BGL derivative for expression of transporterA in DSM1313	This work
pHK-*transporterB*	pHK-P_{2638}-BGL derivative for expression of transporterB in DSM1313	This work
pHK-*2554*	pHK-P_{2638}-BGL derivative for expression of *2554* in DSM1313	This work
pHK-TT1A	pHK derivative, *E. coli*-*C. thermocellum* shuttle vector, Tel3c/Tel4c RT targetron cassette, *groEl* promoter, CmR/TmR	(3)
pHK-nbdA366a	pHK-TT1A derivative, nbdA366a intron	This work
pHK-cbpB32a	pHK-TT1A derivative, cbpB32a intron	This work
pHK-cbpC180a	pHK-TT1A derivative, cbpC180a intron	This work
pHK-cbpD190a	pHK-TT1A derivative, cbpD190a intron	This work
pHK-lbp58a	pHK-TT1A derivative, lbp58a intron	This work

pHK-2554_59a	pHK-TT1A derivative, 2554_59a intron	This work
pHK-HR-Δ*transporterA*	pHK derivative, containing *pyrF* cassette, *tdk* cassette, and homology regions for *transporterA* deletion	This work
pHK-HR-Δ*transporterB*	pHK derivative, containing *pyrF* cassette, *tdk* cassette, and homology regions for *transporterB* deletion	This work
pHK-HR-Δ*2554*	pHK derivative, containing *pyrF* cassette, *tdk* cassette, and homology regions for *clo1313_2554* deletion	This work

References

1. Zhang J, Liu S, Li R, Hong W, Xiao Y, Feng Y, Cui Q, Liu Y-J. 2017. Efficient whole-cell-catalyzing cellulose saccharification using engineered *Clostridium thermocellum*. Biotechnol Biofuels 10:124.
2. Qi K, Chen C, Yan F, Feng Y, Bayer EA, Kosugi A, Cui Q, Liu YJ. 2021. Coordinated β-glucosidase activity with the cellulosome is effective for enhanced lignocellulose saccharification. Bioresour Technol 337:125441.
3. Wei H, Fu Y, Magnusson L, Baker JO, Maness PC, Xu Q, Yang S, Bowersox A, Bogorad I, Wang W, Tucker MP, Himmel ME, Ding SY. 2014. Comparison of transcriptional profiles of *Clostridium thermocellum* grown on cellobiose and pretreated yellow poplar using RNA-Seq. Front Microbiol 5:142.
