# Supplementary material for: Deciphering Cellodextrin and Glucose Uptake in Clostridium thermocellum
Source: mBio. 2022 Sep 7;13(5):e01476-22. doi: 10.1128/mbio.01476-22 (PMC9601137; doi:10.1128/mbio.01476-22)
Supplement: TABLE S3 [file mbio.01476-22-s0008.pdf]

**Table S3.** Primers used in this study. Underlined sequences indicate the restriction sites.

| Primers | Sequence (5'-3') | Usage/comments |
|---|---|---|
| *A*-F | TCTACAGAGTTATTTTTATTTCTAGATTATTTCTTTGGATAGTACTC | To amplify genes to construct plasmids for gene complementation plasmids in *C. thermocellum* |
| A-R | AGCAAGGAGGAGCGTAAATGGAATTCGATAATGCTTTACTTCTGGAAG | |
| B-F | TCCAGAAGTAAAGCATTATCGAATTCCATAGTTTTTTTCCCCCTTT | |
| B-R | TATGCTTCCGGCTCGTATGTGCTAGCTTAATATGCCGACCACGTTG | |
| 2554-F | AGAGTTATTTTTATTTCTAGATTAGTTCATTATTGTTTTTTCAGTTTCC | |
| 2554-R | GAGGAATTTGTTATGGAATTCGCAAGCGTTAAACTTAAAGGTG | |
| UNI | TAACGAGGCTTCTAGCG | To construct targetrons for gene inactivation in *C. thermocellum* |
| nbdA366a-IBS12 | AAAACTAGTAAGTATCGCATCGGCGTGCGACGCGAAAGCTAG | |
| nbdA366a-IBS2s | CGCTAGAAGCCTCGTTAGATACAGCAGGCCAAAGATGCTG | |
| nbdA366a-IBS1a | CCCCGTACGCTGAATCGGCAGCAGCGTATCCAATCC | |
| cbpB32a-IBS12 | AAAACTAGTAAAGCCATAACAGCAGTGCGACGCGAAAGCTAG | |
| cbpB32a-IBS2s | CGCTAGAAGCCTCGTTATGGCTAGCAGGCCAAAGATGCTG | |
| cbpB32a -IBS1a | CCCCGTACGCTGAACAGCAAGCAGCGTATCCAATCC | |
| cbpC180a-IBS12 | AAAACTAGTAAACTCCACATTTGGGTGCGACGCGAAAGCTAG | |
| cbpC180a-IBS2s | CGCTAGAAGCCTCGTTAGGAGTAGCAGGCCAAAGATGCTG | |
| cbpC180a-IBS1a | CCCCGTACGCTGAATTTGGAGCAGCGTATCCAATCC | |
| cbpD190a-IBS12 | AAAACTAGTAATTGGTGTCAGCCAGTGCGACGCGAAAGCTAG | |
| cbpD190a-IBS2s | CGCTAGAAGCCTCGTTAACCAAAGCAGGCCAAAGATGCTG | |
| cbpD190a-IBS1a | CCCCGTACGCTGACAGCCAAGCAGCGTATCCAATCC | |
| lbp58a-IBS12 | AAAACTAGTAATTTTTTCCGCCACGTGCGACGCGAAAGCTAG | |
| lbp58a-IBS2s | CGCTAGAAGCCTCGTTAAAAAAAGCAGGCCAAAGATGCTG | |
| lbp58a-IBS1a | CCCCGTACGCTGACGCCACAGCAGCGTATCCAATCC | |
| 2554_59a-IBS12 | AGCCAAAGCAGGTTGACTAGTAAATTAAAGTCGTTTGTGCGACGCGAAAGCTAG | |
| 2554_59a-IBS2s | CGCTAGAAGCCTCGTTATTAATAGCAGGCCAAAGATGCTG | |
| 2554_59a-IBS1a | GCGGAGTTGCTGTCCCCGTACGCTGATCGTTTAGCAGCGTATCCAATCC | |
| A-up-F | CACTGATTAAGCATTGGTAATCTAGAGCAAAGGAAATAAACAGAGCAG | To amplify upstream and downstream |

| | | |
|---|---|---|
| A-up-R | TAATATACACTTCCATTCAGGTCGACTTAGTTACTTGACAGAAAATCAATATTAT | homology regions to construct plasmids for gene deletion |
| A-down-F | CGTTGCGAGACAGGAAGTAACTCGAGGAATAAAATAAAATCAGCATGAAAG | |
| A-down-R | GAGTAAACTTGGTCTGACAGGAATTCTCGTACATCTCATCAATCGTTGC | |
| B-up-F | CACTGATTAAGCATTGGTAATCTAGAAAGATTTGAACAACATAATG | |
| B-up-R | TAATATACACTTCCATTCAGGTCGACTTCATATGAGCTGCATATTAATTATG | |
| B-down-F | CGTTGCGAGACAGGAAGTAACTCGAGGTTTGACGGACAGATAGAGAGAT | |
| B-down-R | GTAAACTTGGTCTGACAGGAATTCTCAGCAACAAATCTCATTGG | |
| 2554-up-F | AATTTCTCTGAAACCCCTATATTTCGGCCGTTCTTTCCCTCCCAATTATTTTAC | |
| 2554-up-R | AATTTCTCTGAAACCCCTATATTTCGGCCGTTCTTTCCCTCCCAATTATTTTAC | |
| 2554-down-F | CGTTGCGAGACAGGAAGTAACTCGAGAAATATAGGGGTTTCAGAGAAATT | |
| 2554-down-R | GAGTAAACTTGGTCTGACAGGAATTCTTTGTTAATCGTATACATCGTCTC | |
| TT1A-F | ATGCATGTTTGATAACGAAAAG | Sequencing of targetron plasmids and transformants |
| TT1A-R | CGTCATATGTATATCTCCTTC | |
| pHK-F | TGAAATACCGCACAGATGCG | Sequencing of plasmids and transformants for gene complementation |
| pHK-R | GGGCTGGCACGACAGGTTTC | |
| HR-F | TCAGGCAACTATGGATGAAC | Sequencing of plasmids and transformants for gene deletion |
| HR-R | GCTCAGTGGAACGAAAACTC | |
| nbdA366a-F | AAAGTTGGTAAAGCAAGGAGG | Sequencing of gene inactivation mutants |
| nbdA366a-R | TATTTCCACAAGTTGCTGTTCAC | |
| cbpB32a-F | CATTAAAGGGGGAAAAAAACT | |
| cbpB32a-R | CTTTTCTGTAGAAGAGAGCTC | |
| cbpC180a-F | ATGAAAAAATTTATAACAATG | |
| cbpC180a-R | GAGCTGCGTCTTTAGCAGAC | |
| cbpD190a-F | ATGTTAAAGTTAAAAAAAGT | |
| cbpD190a-R | CAACTTTTTCACCGTCAATC | |
| lbp58a-F | CGCCCTTGGAATTATCTAAC | |
| lbp58a-R | GGAGCATAGTCGGCATCGGT | |
| 2554_59a-F | GGCAAGCGTTAAACTTAAAGG | |
| 2554_59a-R | TCTGAGTTTTGCGTCAAGGT | |
| ΔA-F | TGGTCCCGGACAGGTTTTTC | Sequencing of gene deleting mutants |
| ΔA-R | CGTCCACCTCATTCTTTGCGC | |
| ΔB- F | AACCTTGGTTCGGGAACTGC | |
| ΔB- R | GCCTACATTCTCGCCGAAGG | |
| Δ2554-F | GTTTGCCTTCATCCAGTATCC | |
| Δ2554-R | TTCACTTACATTAAGATTGTTTTC | |
| CbpA-F | ACTGGATCCGCAAAAGGCTATGTAGGCGATCC | To construct pET28a-SMT3 plasmids for |
| CbpA-R | GCGCTCGAGTTATTTCTTTGGATAGTACTC | |
| CbpB-F | ACTGGATCCGTAAAATTGACCATGTGGATC | |

| | | |
|---|---|---|
| CbpB-R | GCGCTCGAGTTATTTGCTTGTTTCTTGC | protein expression and purification in *E. coli* |
| CbpC-F | CTGGATCCTCTGGCAGCCGGGATGACACG | |
| CbpC-R | GCGCTCGAGTTACTCATTTAAAAACAAATTTACTTTTTGCTG | |
| CbpD-F | ACTGGATCCGTGTTAGTTGGATGTGGAAAC | |
| CbpD-R | GCGCTCGAGTTAATTTGCCTCTCCATATAAC | |
| Lbp-F | ACTGGATCCAAAAATTCCGGCAACAACAGC | |
| Lbp-R | GCGCTCGAGTTAGTCGTCTAATTCGTATATGTTGC | |
| Clo1313_2554-F | TAAGAAGGAGATATACATATGGCAAGCGTTAAACTTAA | |
| Clo1313_2554-R | GTGGTGGTGGTGGTGCTCGAGGTTCATTATTGTTTTTTCAG | |
